# Integrated approach to generate artificial samples with low tumor fraction for somatic variant calling benchmarking

**DOI:** 10.1186/s12859-024-05793-8

**Published:** 2024-05-08

**Authors:** Aldo Sergi, Luca Beltrame, Sergio Marchini, Marco Masseroli

**Affiliations:** 1 Dipartimento di Elettronica, Informazione e Bioingegneria, Politecnico di Milano, Via Ponzio 34/5, 20133 Milan, Italy; 2 IRCCS Humanitas Research Hospital, Via Manzoni 56, 20089 Milan, Rozzano Italy

**Keywords:** Bioinformatics, DNA sequencing, Low-fraction variant calling, Somatic variant analysis

## Abstract

**Background:**

High-throughput sequencing (HTS) has become the gold standard approach for variant analysis in cancer research. However, somatic variants may occur at low fractions due to contamination from normal cells or tumor heterogeneity; this poses a significant challenge for standard HTS analysis pipelines. The problem is exacerbated in scenarios with minimal tumor DNA, such as circulating tumor DNA in plasma. Assessing sensitivity and detection of HTS approaches in such cases is paramount, but time-consuming and expensive: specialized experimental protocols and a sufficient quantity of samples are required for processing and analysis. To overcome these limitations, we propose a new computational approach specifically designed for the generation of artificial datasets suitable for this task, simulating ultra-deep targeted sequencing data with low-fraction variants and demonstrating their effectiveness in benchmarking low-fraction variant calling.

**Results:**

Our approach enables the generation of artificial raw reads that mimic real data without relying on pre-existing data by using NEAT, a fine-grained read simulator that generates artificial datasets using models learned from multiple different datasets. Then, it incorporates low-fraction variants to simulate somatic mutations in samples with minimal tumor DNA content. To prove the suitability of the created artificial datasets for low-fraction variant calling benchmarking, we used them as ground truth to evaluate the performance of widely-used variant calling algorithms: they allowed us to define tuned parameter values of major variant callers, considerably improving their detection of very low-fraction variants.

**Conclusions:**

Our findings highlight both the pivotal role of our approach in creating adequate artificial datasets with low tumor fraction, facilitating rapid prototyping and benchmarking of algorithms for such dataset type, as well as the important need of advancing low-fraction variant calling techniques.

**Supplementary Information:**

The online version contains supplementary material available at 10.1186/s12859-024-05793-8.

## Background

High-throughput sequencing (HTS) has been extensively used to characterize genomic alterations in tumors through the identification of single nucleotide variants [1–3], copy number alterations [4, 5] and large genomic rearrangements [6]. Moreover, HTS techniques have been used to study tumor subclones, which are genetically distinct subpopulations within a tumor mass that can lead to therapy resistance and disease recurrence.

Recently, HTS has also been used to identify and characterize tumor-derived alterations present in biological fluids, such as circulating tumor DNA (ctDNA) in plasma samples; this opens new avenues for future developments of novel diagnostic and prognostic tools, and paves the way towards the development of personalized treatment strategies for cancer patients [7–12]. ctDNA analysis offers several advantages over traditional biopsy techniques. Unlike invasive tissue sampling, ctDNA analysis involves the detection and analysis of DNA fragments shed by tumor cells into the bloodstream, making it a non-invasive and less risky approach [13].

Furthermore, ctDNA analysis can provide a more comprehensive view of a tumor genetic makeup, as it allows the detection of variants and other genetic changes in multiple regions of the genome [9, 14]. This can be particularly useful for tracking the emergence of tumor cells with improved fitness (such as resistance to treatment), and for monitoring the effectiveness of therapies over time [9, 11, 13]. Although current analysis methods for HTS data are fairly mature, their use in these kinds of samples is challenging, due to the low tumor content compared to non-tumor content [9, 15]. This is particularly problematic for somatic variants with low fractions, typically less than 5%, which often fall below the detection limit of many variant callers.

Variability in library preparation, sequencing, read alignment, and variant calling further complicates the analysis and can introduce biases and false positive variants [16]. To address these challenges, different approaches have been developed, such as ultra-deep sequencing [17] and the use of unique molecular identifiers (UMIs) [11, 18, 19]. Ultra-deep sequencing provides high sequencing coverage (10,000× or higher), enabling the detection of rare or low-abundance DNA sequences. However, the cost of this technique limits its widespread use in ctDNA analysis. UMIs, on the other hand, reduce error rates by identifying and removing duplicate reads [11]. However, they can introduce bias and interfere with amplification [20], reducing assay sensitivity.

While there has been noticeable progress on the experimental side towards addressing these issues, analysis methods have not improved with the same speed, and important challenges, mostly related to sensitivity, still persist. To overcome these challenges, it is important to provide accurate benchmarking of analysis algorithms suited for the specific biological problem. Thus, to save experiment time and costs, artificial datasets are often used for evaluating different analysis strategies. Consortia, like the Genome in a Bottle (GIAB) [21], make artificial datasets available; alternatively, artificial HTS data can be either constructed from scratch with tools like ART [22] or the NExt-generation sequencing Analysis Toolkit (NEAT) [23], or variants can be spiked in existing HTS data with programs like BAMSurgeon [24]. However, despite prior use of artificial data for benchmarking [25, 26], most evaluation approaches rely on modifying existing real experimental data, which limits them to the availability of at least one real data sample with the required features (e.g., library type and coverage). In particular, most of HTS data available are from whole genome sequencing (WGS) or whole exome sequencing (WES) and, as a consequence, the majority of artificial HTS data reflects these types of experiments. Thus, benchmarking variant calling tools for targeted ultra-deep sequencing (as opposed to WES or WGS) is still a challenge. Despite a number of papers have been published on benchmarking somatic variant callers [27–30], to our knowledge none of them addresses low-fraction variants, not having available data samples adequate for such variants.

In this work, we propose a novel approach to generate artificial datasets suitable to simulate ultra-deep targeted sequencing experiments, and subsequently benchmark multiple variant calling algorithms on the generated artificial datasets. Our method takes advantage of the capability of NEAT, a fine-grained read simulator that generates data from scratch based on models learned from different datasets, and then integrates the generated datasets with spike-ins of randomly generated somatic variants; this allows producing artificial somatic tumor datasets with a specified variant allele fraction (VAF), on which variant callers can be reliably tested and benchmarked. Thanks to these artificial datasets, we were able to benchmark typical variant calling algorithms using their default parameters against low- and very low-fraction (< 5%) variants, highlighting a number of limitations. To address them, we evaluated and selected optimal parameter combinations for each variant calling algorithm. Ultimately, our approach provides a significant improvement for the generation of artificial data sets, as a companion to efforts to improve existing computational methods for the identification and study of low-fraction variants.

## Implementation

**Fig. 1:**
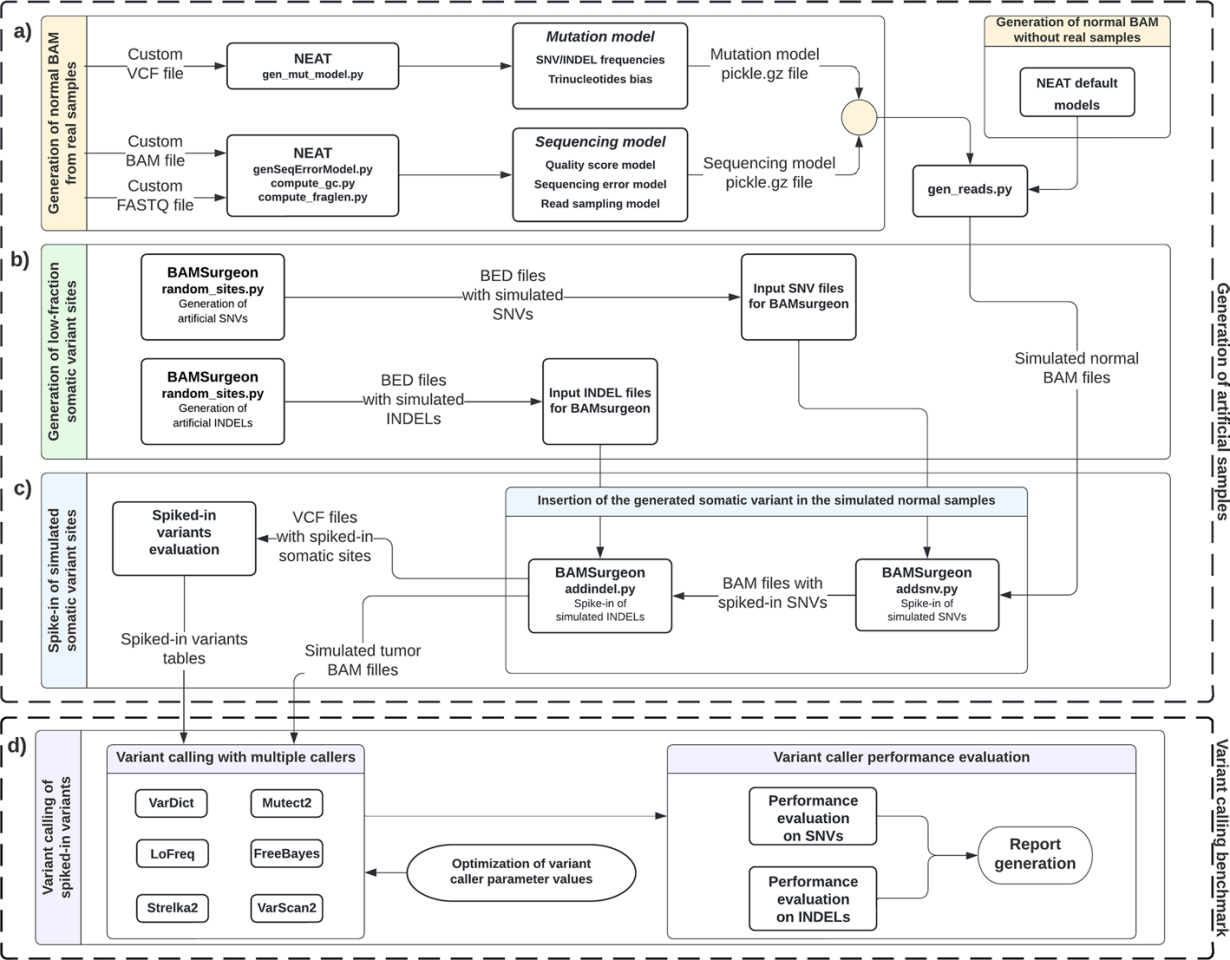
**Flowchart of our approach for the generation of artificial datasets, and their use for low-fraction variant calling tuning and benchmarking.** **a** Artificial normal BAM files are generated either from a real biological sample or from scratch, using mutation and sequencing models. **b** In parallel, low-fraction somatic variants (single nucleotide variants—SNVs and insertions/deletions—INDELs) are generated and Browser Extensible Data (BED) files containing the genomic coordinates of the simulated variant sites are produced. **c** The created BED files are used as input to spike-in the simulated variants (both SNVs and INDELs) in the artificial normal BAM files generated in the first step of our approach. Two types of output are produced: a VCF file containing the successfully spiked-in variants and a BAM file, spiked-in with the simulated somatic variants; the former one can then be used to establish the ground truth on which variant calling performance is evaluated, while the latter one can be fed into several variant callers in **d** to study the calibration of their parameter values and evaluate their performance, comparing the ground truth with VCF files in output from each variant caller. Lastly, a report containing the summarized performance is generated. Step d) can be run independently to perform variant calling and benchmarking also on user-provided pre-existing BAM files

Our approach is composed of multiple steps (Fig. 1). Firstly, a series of artificial normal data samples is generated using both a mutation model, which simulates a baseline mutational rate in the sample, and a sequencing model, which simulates errors from the sequencing technology. Subsequently, for each sample, a unique set of somatic variants (single nucleotide variants—SNVs and insertions-deletions—INDELs) is randomly generated; these variants are then randomly spiked in the generated normal samples at specified variant allele fractions, obtaining artificial tumor data samples. Finally, the latter ones can be used to evaluate, tune and benchmark commonly used variant calling algorithms, testing their ability to identify the somatic variants spiked-in at low- and very low-variant allele fractions. These steps are described as follows, and additional details are provided in Additional file 1: Section S1.

### Creation of artificial normal data samples

The initial step of our approach involves the generation of artificial normal DNA sequence reads, without the need of existing data as a template.

The NEAT software [23] is used to generate the desired number of artificial normal data samples, covering either the whole genome or specific user-supplied genomic regions of interest. In order to take into account systematic errors caused by sequencing instruments, a sequencing error model (describing GC coverage bias distribution, empirical fragment length distribution, and sequence errors) is used to mimic sequencer errors and artifacts. This model can be generated either from user-supplied real data, or from the NEAT default model (i.e., a paired-end sequencing error model generated from the publicly available NA24694 cell line).

Then, a mutational background representative of a normal, non-tumor sample is generated. For this purpose, a mutational model is used, based either on real data in input from the user or on a default model already available from NEAT (e.g., the gold standard for variant evaluation, i.e., the B-lymphocyte derived NA12878 cell line [31]).

Lastly, artificial data samples are generated with any desired level of coverage (e.g., 20,000×).

### Generation of artificial somatic low-fraction variants and their spike-in

The desired number of somatic variants (both SNVs and INDELs) is randomly generated by supplying a defined range of VAF (e.g., 10–0.5%) to the *random_sites.py* script, provided by BAMSurgeon [24].

The somatic variants thus generated are then spiked in the artificial, non-tumor, control Binary Alignment Mapping (BAM) files previously generated with NEAT. SNVs and INDELs are inserted separately in two distinct steps. SNVs can be inserted with a certain minimum and maximum read depth, set equal to the sample coverage in order to correctly generate the variants.

After spiking-in the SNVs, a comparison is run between the originally generated variants and the actual variants successfully inserted. This allows checking spiking-in variability due to both the way variants are inserted [24] and the sequence features of the regions where spiking-in occurs (e.g., low complexity, repeats, presence of existing SNPs), as these features may prevent the designed variants from being correctly inserted. The performance of this process is assessed through the number of successful spike-in events.

Subsequently, the INDELs are additionally spiked in, using the *add_indel.py* script provided by BAMsurgeon. Process performance assessment is done in the same manner as for SNVs.

### Variant calling, tuning and benchmarking

Two different variant calling approaches, which mimic those used by the best practices in the bioinformatics community, are used to evaluate several well-known variant callers (Table 1).

**Table 1:** Variant callers benchmarked in this study

Variant caller	Type of analysis	Core algorithm	Version	Reference
VarDict	Tumor-only, Paired	Heuristic threshold	1.8.2	[32]
MuTect2	Tumor-only, Paired	Haplotype analysis	4.2.3.0	[33]
LoFreq	Tumor-only, Paired	Allele frequency analysis	2.1.5	[34]
VarScan2	Tumor-only, Paired	Heuristic threshold	2.4.4	[35]
FreeBayes	Tumor-only, Paired	Haplotype analysis	1.3.6	[36]
Strelka2	Paired	Allele frequency analysis	2.9.10	[37]

The first approach (tumor-only) uses the artificial tumor data samples only to perform the variant calling. Then, variant calling performances are evaluated using the spiked-in variants as ground truth, while non-tumor variants generated in the normal data samples are not considered for the assessment and filtered out if called. The second approach (paired) uses a tumor-normal paired analysis: each tumor data sample is fed to the variant callers along with a non-tumor data sample (in this case, the data sample before spiking-in) in order to remove non-tumor variants, replicating the same approach used with real data samples.

Performance evaluation is performed with three distinct approaches for each variant caller: firstly, using the default parameter values, or those commonly used in the best practices [16]; secondly, removing the limit of detection from each variant caller, and lastly, using variant calling parameter values tuned to further increase sensitivity (and precision) in low-fraction variant calling (Additional file 1: Section S1.4).

Variants called by each variant caller are compared to the ground truth variants that were successfully spiked-in (which are contained in the Variant Call Format (VCF) files generated by BAMsurgeon). True Positive (TP), False Positive (FP) and False Negative (FN) called variants are evaluated for each sample and used to calculate the True Positive Rate (TPR), Positive Predictive Value (PPV) and False Discovery Rate (FDR) (Additional file 1: Section S1.5). These metrics are then used to evaluate, for each approach used, the performance of every variant caller on low-fraction variants.

#### Variant calling tuning

To perform the variant calling parameter tuning, tumor samples are divided in two subsets: the training set, composed of a total of 14 samples: 7 with spiked SNVs only, and 7 with both SNVs and INDELs spiked in; the test set, composed of 6 samples: 3 with SNVs only, and 3 with both SNVs and INDELs.

The training set is used to systematically evaluate the candidate parameter values, both alone and in combination; the purpose is to first assess if the proposed values can improve the variant calling performance (sensitivity and precision) and, then, to detect the set of parameter values that provides the best performance. The test set is then used to confirm the performance of the chosen set of parameter values, in order to avoid possible overfitting and ensure robustness. Candidate parameter values are chosen through careful review of the variant calling software documentation and discussion with experts.

### Downsampling of coverage depth

To assess the impact of coverage depth on variant calling performance, BAM files are downsampled by systematically varying the percentage of BAM reads from 2 to 80%, with a stepwise increment of 2% for the 2–8% range and 20% for the 20–80% range. To select the reads for downsampling, a random selection is employed, with the random seed set to a fixed value for each subsampling iteration to ensure reproducibility. This minimizes biases in the downsampled data towards ensuring that a representative subset of reads is retained for downstream analyses.

### Generation of DREAM-challenge comparable dataset

To ensure robust validation of our approach, we generated ten artificial samples with characteristics identical to those of the DREAM challenge datasets [24], featuring coverage of 30X, read length of 101 and aligned to the GRCh37 reference genome. To match the DREAM datasets, we introduced 100 random SNVs into each artificial sample, with VAF values ranging from 0.3 to 0.5—consistently with the VAFs in the DREAM datasets [24]. The variant calling performances obtained with these samples were then compared against those of the DREAM challenge set 1 normal dataset [38], in which we introduced 100 random SNVs with identical VAF range values (0.3–0.5).

### Workflow standardization

The entire method has been formalized and standardized in a workflow, named LFV-benchmark, implemented using Nextflow [39]; it is publicly available at https://github.com/DIncalciLab/LFV-benchmark together with its full documentation to test and run the pipeline, and along with different use cases and their example testing datasets. The workflow is completely automated and parallelized, and each step of the workflow can be executed separately, allowing the user to reproduce each step independently (e.g., only generating the artificial samples, or just running the variant calling benchmark on the created artificial samples or on available samples).

## Results

In order to validate our approach and its implementation, we tested our entire workflow by generating multiple artificial samples with different types of low-fraction somatic variants, and using them to tune and benchmark multiple variant calling algorithms for the identification of such variants. In particular, we simulated a scenario where low-fraction variant calling is routinely needed: the analysis of samples from ctDNA, where the percentage of actual tumor DNA is low compared to the total DNA, leading to a low expected fraction of somatic variants.

### Creation of an artificial normal dataset

We firstly constructed 10 artificial normal samples at high coverage, i.e., 30,000×, a common sequencing depth to identify low-fraction variants in circulating tumor DNA. We built the artificial reads with the assumption of a baseline mutation rate expected in normal samples; thus, we relied on the mutational model based on the Platinum Genome NA12878 cell line [31] to simulate a total of 2975 germline variants, which were inserted in the artificial samples with a variant allele fraction of 50%. Out of these inserted variants, 2655 (89.24%) were SNVs, 155 (5.21%) insertions and 165 (5.55%) deletions.

### Generation of artificial somatic low-fraction variants and of an artificial tumor dataset

We randomly generated 1000 SNVs and 1000 INDELs (with a maximum length of 90 bp), covering the TP53, BRCA1 and BRCA2 genes (chosen arbitrarily as an example of a gene panel for ultra-deep sequencing); furthermore, we also randomly generated 1,000 short INDELs (max length 3 bp), to evaluate the performance of variant callers with varying INDEL size. The VAF ranged from 5.0 to 0.1%, 0.08 and 0.1% for SNVs, INDELs and short INDELs, respectively.

To construct an artificial tumor dataset with low-fraction somatic variants, 100 of the 1,000 generated SNVs were spiked in each of the 10 created artificial normal samples, with the same coverage as the normal dataset (30,000×). We verified the reliability of the spike-in process by comparing the actual inserted variants versus the ones used for the spike-in: 997 out of 1000 somatic SNVs were correctly inserted. At most, only one SNV per sample was not inserted (Additional file 1: Table 1). To confirm that the spike-in process did not alter the VAF of the somatic variants, we compared the VAF distribution of the expected (generated) versus the observed (spiked-in) SNVs. VAF distributions for the entire artificial sample cohort (Additional file 1: Figure 1) showed that the spike-in process of the SNVs did not significantly alter the VAF distribution (Kolmogorov-Smirnov test,* p*-value = 0.99). The maximum and minimum VAF for spiked-in SNVs were 4.8 and 0.1%, respectively (Additional file 1: Figure 1), in line with those of the generated SNVs, confirming that the spike-in process of the SNVs does not alter their VAF.

Secondly, 100 of the generated 1000 INDELs or short INDELs were spiked in each of the created artificial normal samples, with the same maximum depth as the SNVs. 100% of the INDELs and short INDELs were spiked in each sample (Additional file 1: Tables 2 and 3). Their VAF distributions for the entire artificial sample cohort are reported in Additional file 1: Figures 3 and 4, for INDELs and short INDELs, respectively. VAF values ranged between 4.7 and 0.08% for INDELs and between 4.9 and 0.1% for short INDELs. Similarly to SNVs, the spike-in process appears not to significantly alter the distribution of VAF for INDELs (Kolmogorov-Smirnov test* p*-value: 0.16 and 0.91 for INDELs and short INDELs, respectively).

These results show that the generation and spike-in of all the artificial variants designed in our method and implemented in the provided workflow were correctly performed; thus, the generated dataset of artificial tumor samples can be used for downstream variant caller benchmarking.

### Variant calling performance evaluation on low-fraction variants

**Table 2:** Performance of variant callers in tumor-normal paired mode and tumor-only mode, when run with their parameter default values, without the limit of detection, or with the tuned parameter values, on samples containing only SNVs

Parameter values	Variant calling mode	Dataset	VarDict		Mutect2		LoFreq		VarScan2		FreeBayes		Strelka2	
			*TPR*	*PPV*	*TPR*	*PPV*	*TPR*	*PPV*	*TPR*	*PPV*	*TPR*	*PPV*	*TPR*	*PPV*
Default	Tumor-normal paired	Whole cohort	**0**.**684**	**0**.**984**	**0**.**373**	0.981	**0**.**220**	0.982	N.A.	N.A.	N.A.	N.A.	**0**.**700**	**0**.**913**
	Tumor-only		**0**.**685**	**0**.**763**	**0**.**643**	0.754	**0**.**796**	**0**.**790**	N.A.	N.A.	N.A.	N.A.	N.A.	N.A.
No detection limit	Tumor-normal paired	Whole cohort	**0**.**833**	**0**.**986**	0.373	0.981	N.A.	N.A.	**0**.**195**	**0**.**979**	**0**.**682**	**0**.**770**	N.A.	N.A.
	Tumor-only		**0**.**835**	**0**.**797**	0.643	0.754	N.A.	N.A.	**0**.**884**	**0**.**010**	**0**.**682**	**0**.**770**	N.A.	N.A.
Tuned	Tumor-normal paired	Training set	0.862	0.955	0.738	0.984	0.240	0.982	0.323	1.0	N.A.	N.A.	0.720	0.935
		Test set	**0**.**862**	0.955	**0**.**741**	0.986	**0**.**258**	0.987	0.117	1.0	N.A.	N.A.	N.A.	N.A.
	Tumor-only	Training set	0.835	0.797	0.781	0.784	0.756	0.986	0.905	0.020	N.A.	N.A.	N.A.	N.A.
		Test set	**0**.**865**	0.791	**0**.**791**	0.800	**0**.**825**	0.806	0.892	0.015	N.A.	N.A.	N.A.	N.A.

N.A. = Not Applicable. Values reported in the text are in bold

The artificial tumor data obtained after the spike-in process was fed to widely used variant callers in order to test their performance in detecting low-fraction variants (SNVs or/and INDELs). The generated artificial germline variants that were called were then excluded from the evaluation, since they represent the normal mutational background and have a much higher VAF. Likewise, when evaluating the performance of calling individual types of variants (SNVs or INDELs), only the specific variant type under analysis was considered.

Performance of variant callers was evaluated in three different scenarios: first, calling algorithms were tested under their standard conditions, i.e., when run using their parameter default values (with the exception of VarScan2 and FreeBayes whose limit of detection was too high, see Additional file 1: Section S1.4 for details); then, their limit of detection (if applicable) was either lowered or removed outright, to evaluate their sensitivity and precision in such condition; lastly, we applied a calibration approach (see Implementation) to systematically test different combinations of parameter values for each variant caller, to assess whether variant calling could be improved at low and very low VAFs without generating too many false positive calls.

#### Performance on samples containing only SNVs

**Table 3:** Performance of variant callers in tumor-normal paired mode and tumor-only mode, when run with their parameter default values, without the limit of detection, or with the tuned parameter values, on samples containing both SNVs and INDELs (max length 90 bp)

Parameter values	Variant calling mode	Dataset	Type of variant	VarDict		Mutect2		LoFreq		VarScan2		FreeBayes		Strelka2	
				*TPR*	*PPV*	*TPR*	*PPV*	*TPR*	*PPV*	*TPR*	*PPV*	*TPR*	*PPV*	*TPR*	*PPV*
Default	Tumor-normal paired	Whole cohort	*SNVs*	0.684	0.925	**0**.**373**	0.981	**0**.**120**	0.975	N.A.	N.A.	N.A.	N.A.	0.700	0.913
			*INDELs*	0.0	0.0	**0**.**198**	**0**.**498**	N.D.	N.D.	N.A.	N.A.	N.A.	N.A.	0.0	0.0
			*SNVs* *+* *INDELs*	0.341	0.399	0.285	**0**.**734**	0.060	0.975	N.A.	N.A.	N.A.	N.A.	0.349	0.911
	Tumor-only	Whole cohort	*SNVs*	0.684	0.727	**0**.**636**	0.752	0.713	**0**.**216**	N.A.	N.A.	N.A.	N.A.	N.A.	N.A.
			*INDELs*	0.0	0.0	**0**.**341**	**0**.**494**	0.0	0.0	N.A.	N.A.	N.A.	N.A.	N.A.	N.A.
			*SNVs* *+* *INDELs*	0.341	0.354	**0**.**489**	0.637	0.356	0.211	N.A.	N.A.	N.A.	N.A.	N.A.	N.A.
No detection limit	Tumor-normal paired	Whole cohort	*SNVs*	0.827	0.885	0.373	0.981	N.A.	N.A.	0.114	**0**.**093**	0.643	**0**.**030**	N.A.	N.A.
			*INDELs*	**0**.**008**	**0**.**0002**	0.198	0.498	N.A.	N.A.	N.D.	N.D.	0.0	0.0	N.A.	N.A.
			*SNVs* *+* *INDELs*	0.417	0.024	0.285	0.734	N.A.	N.A.	0.057	0.093	0.321	0.030	N.A.	N.A.
	Tumor-only	Whole cohort	*SNVs*	0.829	0.728	0.636	0.752	N.A.	N.A.	0.882	0.010	0.643	**0**.**030**	N.A.	N.A.
			*INDELs*	**0**.**011**	**0**.**0001**	0.341	0.494	N.A.	N.A.	N.D.	N.D.	0.0	0.0	N.A.	N.A.
			*SNVs* *+* *INDELs*	0.419	0.013	0.489	0.637	N.A.	N.A.	0.440	0.010	0.321	0.030	N.A.	N.A.
Tuned	Tumor-normal paired	Training set	*SNVs*	N.A.	N.A.	0.745	0.984	N.A.	N.A.	N.A.	N.A.	N.A.	N.A.	N.A.	N.A.
			*INDELs*	N.A.	N.A.	0.311	0.418	N.A.	N.A.	N.A.	N.A.	N.A.	N.A.	N.A.	N.A.
			*SNVs* *+* *INDELs*	N.A.	N.A.	0.528	0.703	N.A.	N.A.	N.A.	N.A.	N.A.	N.A.	N.A.	N.A.
		Test set	*SNVs*	0.859	0.885	**0**.**744**	0.986	0.147	0.977	0.114	0.072	N.A.	N.A.	N.A.	N.A.
			*INDELs*	0.006	0.0001	**0**.**553**	**0**.**734**	N.D.	N.D.	N.D.	N.D.	N.A.	N.A.	N.A.	N.A.
			*SNVs* *+* *INDELs*	0.431	0.020	0.648	0.860	0.073	0.977	0.056	0.072	N.A.	N.A.	N.A.	N.A.
	Tumor-only	Training set	*SNVs*	N.A.	N.A.	0.809	0.790	N.A.	N.A.	N.A.	N.A.	N.A.	N.A.	N.A.	N.A.
			*INDELs*	N.A.	N.A.	0.337	0.407	N.A.	N.A.	N.A.	N.A.	N.A.	N.A.	N.A.	N.A.
			*SNVs* *+* *INDELs*	N.A.	N.A.	0.573	0.619	N.A.	N.A.	N.A.	N.A.	N.A.	N.A.	N.A.	N.A.
		Test set	*SNVs*	0.859	0.742	**0**.**798**	0.801	0.744	0.138	0.889	0.014	N.A.	N.A.	N.A.	N.A.
			*INDELs*	0.006	0.0001	**0**.**590**	**0**.**708**	0.0	0.0	N.D.	N.D.	N.A.	N.A.	N.A.	N.A.
			*SNVs* *+* *INDELs*	0.431	0.013	0.693	0.758	0.371	0.136	0.444	0.014	N.A.	N.A.	N.A.	N.A.

N.A. = Not Applicable; N.D. = Not detected (i.e., no variants were called). Values reported in the text are in bold

Variant calling performances were first evaluated on the artificial samples containing only SNVs spiked-in (Table 2). When the callers were run using their parameter default values, all of them achieved medium to low performance. In tumor-normal paired mode, VarDict and Strelka2 were the most performing callers (Additional file 1: Figure 8), with medium sensitivity (TPR = 0.684 and 0.700, respectively) and high precision (PPV = 0.984 and 0.913, respectively). In tumor-only mode, LoFreq was the most performing one (Additional file 1: Figure 10), for both sensitivity and precision (TPR = 0.796, PPV = 0.790), while VarDict and Mutect2 had limitedly lower performances. Overall, results obtained using the parameter default values show that precision in calling SNVs is high for all variant callers, but their sensitivity is only medium (Additional file 1: Figures 8 and 10).

Then, we evaluated how the calling algorithms perform after disabling their limit of detection (Additional file 1: Figures 12 and  14). LoFreq and Strelka2 were not considered for this evaluation, as they do not provide a parameter to tune such limit. Overall, all algorithms benefit from the removal of their limit of detection in term of sensitivity, with the only exception of Mutect2, whose results were unchanged. VarDict appeared to be the best performing caller: its sensitivity increased for both tumor-normal paired mode (TPR = 0.833 vs. 0.684) and tumor-only mode (TPR = 0.835 vs. 0.685), while its precision did not change significantly in both tumor-normal paired mode (PPV = 0.986 vs. 0.984) and tumor-only mode (PPV = 0.797 vs. 0.763). VarScan2 was able to call variants once its limit of detection was disabled; however, its performance was low: in tumor-normal paired mode it attained high precision but low sensitivity (PPV = 0.979 and TPR = 0.195, respectively), while in tumor-only mode it exhibited high sensitivity (TPR = 0.884) but very low precision (PPV = 0.010). When the detection limit was set below 1%, FreeBayes achieved same medium performance on both tumor-normal paired mode and tumor-only mode (TPR = 0.682 and PPV = 0.770); yet, in this case its runtime was too excessive (several hours for each sample).

Lastly, we evaluated the performance of the variant callers considering different sets of parameter values for each caller, whose best values were chosen through the calibration study described in the Implementation Section. In particular, for each variant caller the considered sets of parameter values were first evaluated on the training set (7 samples with only SNVs spiked-in), and then the best values identified were verified on the test set (3 samples with only SNVs spiked-in).

Results (Table 2, Additional file 1: Figure 21 and 23) showed that the identified best set of parameter values improved the performance of each variant caller, with outcomes in line between training and test sets. The only caller excluded from this analysis was FreeBayes: it did not correctly estimate the VAF, thus it was excluded from any subsequent benchmark.

The highest gains in performance were observed for Mutect2, with significant increases in sensitivity for both tumor-normal paired mode (TPR = 0.741 vs. 0.373, in test set) and tumor-only mode (TPR = 0.791 vs. 0.643, in test set), with no significant changes in the already relevant precision. Also VarDict improved its sensitivity for both tumor-normal paired mode (TPR = 0.862 vs. 0.684, in test set) and tumor-only mode (TPR = 0.865 vs. 0.685, in test set), while keeping its high precision. LoFreq only showed limited changes in sensitivity in both tumor-normal paired mode (TPR = 0.258 vs. 0.220, in test set) and tumor-only mode (TPR = 0.825 vs. 0.796, in test set), while VarScan2 had low performance even after the tuning. Lastly, parameter tuning in Strelka2 did not result in any change in performance compared to when it was run with parameter default values on the same sample subset of the training set (TPR = 0.720 and PPV = 0.935 for both the cases). In light of these findings, Strelka2 was not investigated on the test set.

With regards to SNV calling, these findings show that VarDict and Mutect2 were the most performing ones in both analysis modes (along with LoFreq in tumor-only mode); in particular, Mutect2 showed a much increased sensitivity after parameter tuning, with no loss of precision.

#### Performance on samples containing both SNVs and INDELs

To further evaluate low-fraction variant calling performance on artificial datasets closer to real biological samples, we benchmarked the variant callers on an artificial dataset including both SNVs and INDELs (with maximum length of 90 bp). As the spike-in processes for the two variant types were independent, we assessed the performance of the variant callers on both SNVs and INDELs separately, as well as together. This allowed us to determine whether the presence of INDELs affected the accuracy of SNV calling.

We first evaluated the performance of variant calls after the INDELs spike-in using caller parameter default values. No major changes were observed in SNVs calling performance (Table 3, Additional file 1: Figures 9a and 11a) compared to the SNVs only dataset (Table 2, Additional file 1: Figures 8 and 10); the only exception was LoFreq, which had a significant drop in the already very low sensitivity in tumor-normal paired mode (TPR = 0.120 vs. 0.220) and an even much more pronounced drop in precision in tumor-only mode (PPV = 0.216 vs. 0.790). When taking into account only INDELs (Table  3, Additional file 1: Figures 9b and  11b), only Mutect2 was able to call them, although with low performance (just slightly better in tumor-only mode: TPR = 0.341, PPV = 0.494). Therefore, Mutect2 was also the only one able to correctly call both SNVs and INDELs when considered together, with the highest sensitivity in tumor-only mode (TPR = 0.489) and the best precision in tumor-normal paired mode (PPV = 0.734).

We then performed the same evaluations with the limit of detection of the callers removed. In this case (Table 3, Additional file 1: Figures 13a and 15a), INDELs inclusion did not affect SNVs calling for Mutect2 and VarDict only (see comparison with Table 2, Additional file 1: Figures 12 and 14), while precision considerably dropped for VarScan2 and FreeBayes in tumor-normal paired mode (PPV = 0.093 vs. 0.979 and PPV = 0.030 vs. 0.770, respectively), and for FreeBayes also in tumor-only mode (PPV = 0.030 vs. 0.770). When INDELs calling was considered (Table 3, Additional file 1: Figures 13b and 15b), VarDict kept a very low performance, both in tumor-normal paired mode (TPR = 0.008, PPV = 0.0002) and in tumor-only mode (TPR = 0.011, PPV = 0.0001), while the performance of Mutect2 did not change. Lastly, VarScan2 and FreeBayes were still not able to detect any INDEL, while LoFreq and Strelka2 were not considered in this evaluation as they do not allow to remove the limit of detection.

Thus, also in the case with no limit of detection, overall Mutect2 was still the best performing caller in both tumor-normal paired mode and tumor-only mode (Table 3).

Finally, through the calibration approach described in the Implementation Section, also for the artificial dataset including both SNVs and INDELs we benchmarked the candidate parameter values of each calling algorithm, with the exception of Strelka2 and FreeBayes according to the previous benchmark on the SNVs only dataset. However, in this case we could investigate the parameter values on the entire training set only for Mutect2, while on only one random sample of the training set for the remaining variant callers, due to the high computational cost induced by the presence of INDELs; all callers using the identified tuned parameter values were then evaluated on the entire test set.

Results in the test set (Table 3, Additional file 1: Figures 22 and 24) showed that with regards to SNVs calling Mutect2 had a significant increase in sensitivity, both in tumor-normal paired mode (TPR = 0.744 vs. 0.373) and in tumor-only mode (TPR = 0.798 vs. 0.636), while maintaining equivalent precision. Also with regards to INDELs calling, the tuning significantly increased Mutect2 sensitivity and also precision both in tumor-normal paired mode (TPR = 0.553 vs. 0.198, PPV = 0.734 vs. 0.498) and in tumor-only mode (TPR = 0.590 vs. 0.341, PPV = 0.708 vs. 0.494). As of the other calling algorithms, parameter value tuning only increased SNVs sensitivity for VarDict and slightly for LoFreq, while did not provide any relevant improvement on INDELs calling, with VarDict showing drop in precision when evaluated on both SNVs and INDELs.

These findings show that Mutect2 was not affected by multiple spike-in processes, and that it was the best performing variant caller even when considering SNVs and INDELs together.

#### Evaluation of influence of low-fraction INDEL length on variant calling performance

To investigate whether the length of low-fraction INDELs affects the performance of variant callers, we evaluated the variant calling performance in high-sensitivity settings (i.e., using caller tuned parameter values) on the dataset with both SNVs and short INDELs (max length 3 bp) spiked-in. The only exceptions were FreeBayes and Strelka2, whose parameter values were not tuned: the former one was run by removing the limit of detection, while the latter one was run using its parameter default values.

Insertion of short INDELs with respect to INDELs of general lenght had no impact on variant calling performance, with sensitivity and precision of SNVs and INDELs calling that were overall only marginally modified (see Additional file 1: Figures 22 and 24 vs. Additional file 1: Figures 25 and 26, respectively). This confirms that overall the length of spiked-in INDELs does not affect variant calling performance.

#### Evaluation of influence of the coverage depth on variant calling performance

We also evaluated the variant calling tools on subsampled samples using the high-sensitivity settings (with the exception of FreeBayes and Strelka2). Consistently with the expectations, we observed a significant decrease in sensitivity as coverage depth was reduced (Additional file 1: Figures 27–30). Precision of the tools was also affected, particularly in tumor-only mode and for certain tools such as VarDict and Freebayes. Interestingly, our findings indicate a decrease in precision with increasing coverage depth for VarScan2 and LoFreq in tumor-only mode. Notably, this behavior was observed only for the dataset with SNVs and INDELs in the case of LoFreq. Furthermore, the sensitivity of VarScan2 noticeably decreased when applied to the dataset containing both SNVs and INDELs in the tumor-normal paired mode, indicating that caution should be taken when using this tool for high-depth samples. Remarkably, Mutect2 remained the most reliable and stable tool across varying coverage depths.

#### Evaluation of influence of the number of spiked-in variants on variant calling performance

To explore the influence of the variant number on the performance of variant callers, we conducted a sensitivity analysis using a downsampled normal high-coverage sample, reduced to 10,000× for computational efficiency. We systematically introduced varying numbers of SNVs—specifically, 200, 100, 50 and 10 SNVs, respectively—into the original sample. Subsequently, the resulting four tumor samples were processed using high-sensitivity settings and tumor-normal paired mode. The outcomes, depicted in Additional file 1: Figure 31, reveal a consistent decrease in both sensitivity and precision as the number of inserted variants increases. Notably, Mutect2 and Strelka2 exhibited exceptional performance in both sensitivity and precision when only 10 variants were introduced, but their performance declined to medium levels for sensitivity (TPR = 0.638 and 0.608 for Mutect2 and Strelka2, respectively) and medium-high levels for precision (PPV = 0.894 and 0.870 for Mutect2 and Strelka2, respectively) as the number of variants increased. VarScan2 and Lofreq experienced a lower decline in performance, although their overall performance remained relatively low. Lastly, VarDict and FreeBayes were excluded from the evaluation due to the extensive computational time they required. Overall, these findings emphasize the need of caution when dealing with an excessively high number of variants, particularly in targeted genomic regions, such as in the case of high-coverage samples.

#### Comparison with the DREAM challenge dataset

To ensure the reliability of our approach in comparison to real samples, we compared the DREAM challenge dataset and generated artificial normal samples with introduced SNVs as outlined in the Implementation section. We conducted a variant calling benchmark in tumor-normal paired mode with high-sensitivity settings enabled. The performance analysis (Additional file 1: Figure 32) consistently demonstrated high and equivalent performance in variant calling output for both the DREAM dataset and the generated samples. Notably, the TPR surpassed 0.7 and remained comparable between the datasets, indicating robust performance. Moreover, precision exceeded 0.9, validating the reliability and consistency of the artificially generated samples. Interestingly, Mutect2 exhibited a decline in performance, with a TPR of 0.597, which may be worth of further investigation in the future. Lastly, FreeBayes and Strelka2 were not able to perform variant calling on the DREAM dataset and were omitted.

## Discussion

In this work we propose an integrated approach, implemented in an automated workflow, which generates specific artificial datasets to benchmark the detection rate of variant callers for low-fraction variants, with minimal human intervention and without the need for real samples as a starting point. The use of artificial datasets to evaluate specific analytical needs for DNA variant detection has been widely employed by the scientific community [24–26]. However, to our knowledge the available approaches rely on the availability of real data to derive artificial samples, which is problematic when evaluations need to be carried out for specific applications.

As it is unfeasible to provide every possible application-specific dataset to the community, a relevant alternative approach is to support the flexible generation of suitable artificial datasets according to the specific experimental and analytical needs. Our method allows the generation of these datasets without a priori experimental data, or alternatively with user-supplied mutational and/or sequencing error models. In addition, the user can specify the genomic regions of interest, making the approach and generated data very useful and suitable for any specific application under study. The method also allows the flexible and reliable insertion of artificial variants, which can be adjusted to match the expected VAF ranges in actual experimental data. In addition, our method includes the evaluation of the generated artificial data with widely-used variant callers, to have a rapid feedback on their performance for the specific use case. It also offers the possibility to run and benchmark the variant callers with or without a matched normal sample (if supported by the caller).

Our results showed that commonly used variant callers have worse performance on very low-fraction variants when run with parameter default values. This is particularly evident when evaluating the calling of INDELs or both SNVs and INDELs in the same dataset (a scenario that matches real samples), with only Mutect2 able to achieve acceptable performance in such cases. When evaluating only SNVs calls, Mutect2 or VarDict are recommended choices, regardless of whether a matched normal sample is available or not. Additionally, also Strelka2 can be used in the case of matched tumor-normal samples.

As using parameter default values the performance of variant callers is suboptimal, leveraging our framework and its generated artificial datasets we performed their calibration for low-fraction variants by adjusting the caller parameter values. Removing the limit of detection (where supported) improved the sensitivity, but at the same time induced or maintained lower precision, especially when INDELs are present.

Even after tuning, performance of most variant callers is suboptimal for low-fraction variants. In particular, acceptable results are produced only in the absence of INDELs, a scenario that is less close to real samples. When INDELs calling is considered, only Mutect2 maintains an acceptable, albeit not high, performance.

Thus, current variant callers are more suitable for calling low-fraction SNVs rather than insertions and deletions. This has important consequences in real-world scenarios, as sequencing library preparations may damage DNA and introduce artifacts, which could negatively affect the calling. A recent study [40] has suggested that other tools, such as Pindel [41], DELLY [42], and DeepVariant [43], may be more appropriate for detecting INDELs. However, their effectiveness in detecting low-fraction variants has not been fully evaluated. While DeepVariant is limited to germline calling, the computational demands of the other tools, including Lancet [44] that has also been shown to perform well in INDELs calling, make challenging to benchmark their performance under the conditions of our study. Further research is needed to evaluate the performance of these tools on low-fraction variants and to optimize their use for accurate variant calling in different sequencing applications.

Given the rise of low-fraction variant evaluations in tumor DNA from plasma or other biological samples, improved and accurate variant calling (as opposed to manual evaluation of variants [45]), capable of discriminating real variants from sequencing and library preparation artifacts, is of utmost importance: either highly optimized parameter values evaluated on large datasets or improved software applications are needed. To this aim, our proposed approach allows generating in a reasonable amount of time a great number of in-silico samples on which variant callers can be tested and benchmarked. Moreover, in-silico datasets can be used not only to benchmark variant callers and rapidly set-up a proper computational workflow for specific experiments, but also to increase the number of available samples to be used in variant analysis.

The main limitation of this approach is that the generated data are of course all artificial; even if there are multiple methods to make them similar to wet lab-generated real sequencing data, they do not perfectly recapitulate the nature of a real experiment. In addition, despite the several datasets publicly available (e.g., the ones from the DREAM challenges [46]), to our knowledge none of them provide sufficient depth and suitable characteristics to benchmark variant calling accuracy for low-fraction variants; thus, they cannot be used either for comparison with our generated artificial datasets. Lastly, our approach does not consider ortogonal means (e.g., annotation-based filtering) or specific experimental strategies (e.g., UMI) to enhance performance. The former can address SNV calling but not INDEL calling, while the latter may introduce biases requiring specific optimizations for our approach. Nevertheless, the approach can be used for rapid prototyping and benchmarking of analytical procedures suited to the biological problem being studied, without the need of actual sequencing; this leads to better designed experiments, more tailored analyses, and more cost-effective sequencing runs.

## Conclusions

We developed a novel approach for generating artificial datasets that can mimic deep and ultra-deep targeted sequencing data, and took advantage of them to tune and benchmark variant calling algorithms. Our approach is useful and flexible, and can be reliably used to simulate various types of data for different experimental needs, thus supporting generalizability. Furthermore, the integrated benchmark of variant calling methods allows effortless evaluations of the performance of most relevant variant callers on the specific data generated. We expect our approach and its implementation to be a relevant and practical contribution to the bioinformatics community towards the design of faster and more precise methods for DNA variant analysis.

### Supplementary Information

**Additional file 1:** Additional information on the code, parameters, high-sensitivity mode, benchmark metrics; additional data and tables on spike-in and variant calling performance.

## Data Availability

LFV-benchmark is freely available at GitHub (https://github.com/DIncalciLab/LFV-benchmark). All information regarding its installation and application is provided.

## Abbreviations

BAM: Binary alignment mapping
BED: Browser extensible data
ctDNA: Circulating tumor DNA
FDR: False discovery rate
FP: False positive
FN: False negative
GIAB: Genome in a bottle
HTS: High-throughput sequencing
INDEL: INsertion-deletion
NEAT: NExt-generation sequencing analysis toolkit
PPV: Positive predictive value
SNV: Single nucleotide variant
TP: True positive
TPR: True positive rate
UMI: Unique molecular identifier
VAF: Variant allele fraction
VCF: Variant call format
WES: Whole exome sequencing
WGS: Whole genome sequencing

## Availability and requirements

Project name: LFV-benchmark. Project home page: https://github.com/DIncalciLab/LFVbenchmark. Operating system(s): POSIX systems (Linux, macOS, etc). Programming language: Python, Bash. Other requirements: Java 11 or higher, Bash 3.2 or higher, Picard, Nextflow. License: MIT. Any restrictions to use by non-academics: None.
