# Supplementary material for: Integrated approach to generate artificial samples with low tumor fraction for somatic variant calling benchmarking
Source: BMC Bioinformatics. 2024 May 8;25:180. doi: 10.1186/s12859-024-05793-8 (PMC11077792; doi:10.1186/s12859-024-05793-8)
Supplement: Supplementary file 1 — Additional file 1: Additional information on the code, parameters, high-sensitivity mode, benchmark metrics; additional data and tables on spike-in and variant calling performance. [file 12859_2024_5793_MOESM1_ESM.pdf]

Supplementary Material of

Integrated approach to generate artificial samples with low tumor fraction for somatic variant calling benchmarking

Aldo Sergi [1,2,*], Luca Beltrame [2], Sergio Marchini [2], Marco Masseroli [1]

[1] Dipartimento di Elettronica, Informazione e Bioingegneria, Politecnico di Milano, via Ponzio 34/5, 20133, Milan, Italy

[2] IRCCS Humanitas Research Hospital, Via Manzoni 56, 20089, Rozzano (MI), Italy

* To whom correspondence should be addressed

S1. Supplementary Implementation

S1.1. Creation of artificial normal datasets

Artificial normal reads are generated with the NExt-generation sequencing Analysis Toolkit (NEAT) version 3.2 (Stephens et al., 2016), using two inputs: a mutational model, which emulates the mutational background normally present in non-tumor samples, and a sequencing model (composed of three sub-models: the quality score model, the sequencing error model and the read sampling model), which mimics artifacts and errors normally generated by the instruments (e.g., sequencers).

The generation of models is described in the NEAT documentation (https://github.com/ncsa/NEAT) and is optional. The process generates two serialized data structures (pickle files): one for the mutational model and one for the sequencing error model. In absence of any of these customized models, the default ones supplied by NEAT are used.

The models are then used to generate reads in Binary Alignment Mapping (BAM) format with the NEAT *gen_reads.py* script as follows:

```
python3 /path/to/NEAT/gen_reads.py -r ${FASTA} \
        -R ${READLEN} \
        --pe-model ${FRAGLENMODEL} \
        -c ${COVERAGE} \
        -e ${SEQERRORMODEL} \
        --gc-model ${GCBIASMODEL} \
        -tr ${BED} \
        --bam \
        --vcf \
        --no-fastq \
        --rng ${RANDOM} \
        -m ${MUTMODEL} \
        -o ${OUTPUT_NAME}
```

where:

- `FASTA` is the reference genome (GRCh38 or hg38)
- `READLEN` is the read length; standard read length in targeted sequencing for Illumina sequencers is 150
- `FRAGLENMODEL` is the fragment length model; we used the default *fraglenModel_default.pickle.gz*
- `COVERAGE` is the expected median coverage for output artificial datasets; we set the value 30,000, a common target in ultra-deep sequencing
- `SEQERRORMODEL` is the sequencing error model; we used the default *errorModel_default.pickle.gz*
- `GCBIASMODEL` is the GC% bias model; we used the default *gcBias_default.pickle.gz*
- `BED` indicates the target regions of interest, e.g., some specific gene regions; it is a tab-separated value text file containing the chromosome names and the genomic positions of the target regions, e.g., constructed from the

genome coordinates for all exons of the genes of interest extracted from the UCSC Table Browser (https://genome.ucsc.edu)

- `--bam` indicates to generate a BAM file in output
- `--vcf` is used to produce a VCF file in output
- `--no-fastq` specifies that no FASTQ files are generated in output, saving space and computational time
- `RANDOM` is the Bash function used to generate a random seed for NEAT
- `MUTMODEL` is the mutational model; we used the *MutModel_NA12878.pickle.gz*, generated from the NA12878 cell line
- `OUTPUT_NAME` is the output name, i.e., the name of the generated files

In addition to the BAM file, using the option `--vcf` also generates the related VCF file containing the random mutations included by NEAT.

S1.2. Generation of low-fraction somatic variants

Somatic mutations, to be spiked in the artificial normal samples from NEAT, are generated using the *random_sites.py* script of the BAMSurgeon suite version 1.3 (Ewing et al., 2015). The script can generate the user-supplied desired number of variants (either single nucleotide variants - SNVs, or insertions-deletions - INDELs), randomly selecting the affected and the varied DNA bases. The allele fraction of the generated variants is randomly sampled from the beta distribution, with the fraction upper and lower bounds selected by the user along with the length of the INDELs.

SNVs are generated on each artificial sample with the following command:

```
python3 random_sites.py -g ${FASTA} \
        -b ${BED} \
        -s ${RANDOM} \
        -n 100 \
        --avoidN \
        --minvaf 0.00001 \
        --maxvaf 0.05 \
        snv > OUTPUT
```

where:

- `FASTA` is the reference genome (GRCh38 or hg38)
- `-b` is the BED file to be fed; it is the same file used for the generation of the normal samples (see section S1.1 for details)
- `RANDOM` is the Bash function used to generate a random seed for *random_sites.py*
- `-n` is the number of random mutations to be generated, e.g., 100
- `--avoidN` is used to avoid regions containing unknown references when spiking-in variants
- `--minvaf` and `--maxvaf` are the minimum and the maximum variant allele fractions (VAF) of the randomly generated mutations, respectively; we set them to 0.00001 (0.001%) and 0.05 (5.0%)
- `snv` specifies the type of variants to be created, i.e., Single Nucleotide Variants (SNV)
- `OUTPUT` is the output file name

INDELs are generated in the same fashion, using the command:

```
python3 random_sites.py -g ${FASTA} \
      -b ${BED} \
      -s ${RANDOM} \
      -n 100 \
      --avoidN \
      --minlen 1 \
      --maxlen 90
      --minvaf 0.00001 \
      --maxvaf 0.05 \
      indel > OUTPUT
```

where, in addition to the above specified parameters:

- `--minlen` and `--maxlen` are the minimum and the maximum base length of the randomly generated INDELs, respectively; we generated two types of INDELs: INDELs of mixed lengths (`--maxlen 90`) and short INDELs (`--maxlen 3`)
- `indel` specifies the type of variants to be created, i.e., insertions/deletions (INDEL)

S1.3. Spike-in of artificial variants

Variants generated with the *random_sites.py* script are then spiked in each artificial normal sample through the scripts *add_snv.py* and *add_indel.py* of the BAMSurgeon suite. SNVs and INDELs are spiked in separately. First, the following command is used to spike-in the somatic SNVs:

```
addsnv.py -v inputbed \
        -f inputbam \
        -r fasta \
        -o output.bam \
        --picardjar PICARDJAR \
        --aligner mem \
        --alignopts c:250,M:,t:8,v:1 \
        -p 8 \
        --ignoresnps \
        --tagreads \
        --mindepth 8 \
        --maxdepth 30000 \
        --tmpdir tmpdir \
        --minmutreads 4 \
        --seed ${RANDOM}
```

where:

- `inputbed` is the BED file containing the SNVs generated in the previous step
- `inputbam` is the artificial BAM file generated by NEAT
- `fasta` is the reference genome (GRCh38 or hg38)
- `output.bam` is the output BAM file name
- `PICARDJAR` is the path to the Picard (https://broadinstitute.github.io/picard/) `.jar` file, required for most aligners
- `mem` indicates the use of the BWA-MEM algorithm (Li and Durbin, 2009) for the alignment process, performed by BAMsurgeon
- `--alignopts` specifies the aligner options, established by the bioinformatics community (https://github.com/bcbio/bcbio-nextgen)
- `-p` splits into the specified number of multiple processes
- `--ignoresnps` forces spiking-in even if the reference allele does not share the relevant reads
- `--tagreads` adds a tag in the generated BAM file ('BS') to the altered reads, to distinguish them from the reads from the original sample (for possible downstream analyses or quality control)
- `--mindepth` and `--maxdepth` are the minimum and maximum read depth to spike-in the mutations
- `--tmpdir` sets the name of the BAMSurgeon temporary directory
- `--minmutreads 4` sets a minimum of four mutated reads
- `RANDOM` is the Bash random function used to create a random seed for BAMSurgeon

INDELs previously generated are spiked-in as done for SNVs, but using the *add_indel.py* script, with the same parameter values. Each type of INDELs generated is inserted separately in the artificial tumor samples previously generated with spiked-in SNVs.

S1.4. Variant calling parameters and their value tuning

Variant calling is performed using six different and widely used variant callers: VarDict (Lai et al., 2016), Mutect2 (Cibulskis et al., 2013), LoFreq (Wilm et al., 2012), VarScan2 (Koboldt et al., 2012), FreeBayes (Garrison and Marth, 2012) and Strelka2 (Kim et al., 2018). We first tested the performance of the variant callers using their default parameter values (default settings), and subsequently we tuned the parameter values of each caller to improve sensitivity (high-

sensitivity settings), including performing a reduction or removal of the minimum variant allele fraction limit. Details about default values of algorithm parameters and their tuning are reported as follows:

VarDict:

Tumor-only mode, default parameters:

```
/path/to/vardict \
        -G FASTA \
        -b TUMOR_BAM \
        -N NAME \
        -f 0.01\
        --nosv \
        -F 0x700 \
        -c 1 -S 2 -E 3 -g 4 \
        BED \
        | teststrandbias.R \
        | var2vcf_valid.pl -N NAME -E \
        > NAME.vcf
```

where:

- `FASTA` is the reference genome （GRCh38 or hg38)
- `TUMOR_BAM` is the input, tumor, BAM file
- `NAME` is the output name of the result
- `0.01` is the default limit of detection of VarDict (i.e., the lowest VAF detectable)
- `--nosv` turns off structural variation calling to save computational time
- `BED` is a tab-separated value text file containing the chromosome names and the genomic positions of the target regions of interest; it is the same file used for the previous step (see Section S1.1 for details)
- `teststrandbias.R` is a R script that removes strand bias (a type of sequencing error)
- `var2vcf_valid.pl` is a PERL script used to remove false positive calls from the output calls
- `-E` (of the `var2vcf_valid.pl` script) does not print the tag `END` at the end of the log when set
- `NAME.vcf` is the output VCF file name

The other parameters are set by default, as stated in the VarDict documentation (https://github.com/AstraZeneca-NGS/VarDictJava#readme).

Tumor-normal paired mode, default parameters:

```
/path/to/vardict \
        -G FASTA \
        -b 'TUMOR_BAM|NORMAL_BAM' \
        -N NAME \
        -f 0.01\
        --nosv \
        -F 0x700 \
        -c 1 -S 2 -E 3 -g 4 \
        BED \
        | testsomatic.R \
        | var2vcf_paired.pl -N 'TUMOR_NAME|NORMAL_NAME' \
        > NAME.vcf
```

where:

- `TUMOR_BAM` is the tumor input BAM file
- `NORMAL_BAM` is the normal, paired, input BAM file
- `testsomatic.R` is the tumor-normal mode equivalent of the `teststrandbias.R` script to remove strand bias (a type of sequencing error)
- `var2vcf_paired.pl` is the tumor-normal mode equivalent of the `var2vcf_valid.pl` script to remove false positive calls from the output calls
- `TUMOR_NAME` and `NORMAL_NAME` are the sample name of the tumor and normal BAM files, respectively

The **limit of detection** is removed by strongly lowering the threshold on the minimum allele fraction, using the following parameter:

- `-f 0.0001`

To detect the best set of parameter values for **high-sensitivity settings**, we first tested the following parameters on a single dataset (with either SNVs or SNVs + INDELs spiked-in) from the training set:

- `-X [default: 2]`: extension of bp to look for mismatches after insertion or deletion
- `--nmfreq [default: 0.1]`: variant frequency threshold to determine variant as good in case of non-monomer Micro Satellite Instability (MSI)
- `--mfreq [default: 0.25]`: variant frequency threshold to determine variant as good in case of monomer MSI
- `-q [default: 22.5]`: phred score for a base to be considered a good call
- `-m [default: 8]`: filter of reads with mismatches more than 8

After the benchmarking results (Supplementary Figure 17), we focused further benchmarks only on the `-q` parameter, which achieved the best performance with the following value:

- `-q 15`

Mutect2:

Tumor-only mode, default parameters:

```
gatk Mutect2 -I TUMOR_BAM\
        --reference FASTA \
        -L BED \
        -O NAME.vcf
```

Tumor-normal paired mode, default parameters:

```
gatk Mutect2 -I TUMOR_BAM \
        -I NORMAL_BAM \
        --normal SAMPLE_NAME_normal
        --reference FASTA \
        -L BED \
        -O NAME.vcf
```

The limit of detection is removed by adding the following parameter:

- `--minimum-allele-fraction 0.0`

To detect the best set of parameter values for **high-sensitivity settings**, we first benchmarked the following parameters individually:

- `---f1r2-max-depth [default: 200]`: groups sites with depth higher than 200
- `--f1r2-median-mq [default: 50]`: sites with median mapping quality below 50 were skipped
- `--max-reads-per-alignment-start [default: 50]`: maximum number of reads to retain per alignment start position. Reads above 50 threshold were downsampled
- `--min-base-quality-score [default: 10]`: minimum base quality required to consider a base for calling
- `--tumor-lod-to-emit [default: 3]`: Log 10 odds threshold to emit variant to VCF

The benchmarking showed that only two of the proposed parameters influenced the software performance on low fraction calling: `max-reads-per-alignment-start` and `min-base-quality-score`. We then benchmarked the performance of Mutect2 when combinations of different values of these two parameters were fed in input to the software (Supplementary Figure 18 and 19). The parameter values that achieved the best performance, in both tumor-normal paired and tumor-only mode, and for both SNVs and INDELs, were:

- `--max-reads-per-alignment-start 100`
- `--min-base-quality-score 25`

LoFreq:

Tumor-only mode, default parameters:

```
lofreq call \
	-f FASTA \
	-o OUTPUT.vcf \
	--call-indels \
	TUMOR_BAM
```

where `--call-indels` is needed to call INDELs, and is set only when INDELs are spiked in the artificial normal samples. Note that when calling INDELs, input BAM files need to be processed using the following command:

```
lofreq indelqual \\
	--dindel \\
	-f ${fasta} \\
	-o OUTPUT_NAME.bam \\
	INPUT_BAM
```

where the `--dindel` command inserts INDEL qualities into the input BAM files and is mandatory for INDELs calling.

Parameter tuning for **high-sensitivity settings** was performed separately for tumor-normal paired mode and tumor-only mode, since the two modes do not share the same commands. For tumor-only mode, the following parameter was benchmarked (Supplementary Figure 20):

- `--sig [default: 0.01]:` p-value cut-off for a variant call

The value that achieved the best performance was:
- `--sig 5`

Tumor-normal paired mode, default parameters:

```
lofreq somatic \
	-n NORMAL_BAM \
	-t TUMOR_BAM \
	-f FASTA \
	-l BED \
	--call-indels \
	-o OUTPUT_NAME
```

To perform parameter tuning for **high-sensitivity settings** for tumor-normal paired mode, following LoFreq documentation (https://csb5.github.io/lofreq/commands/#somatic) we benchmarked two parameters:
- `--tumor-mtc [default: bonf]:` the type of multiple testing correction for tumor; default is the Bonferroni test
- `--tumor-mtc-alpha [default: 1.0]:` the value of the Alpha parameter for the multiple test correction

Likewise the tumor-only mode, these parameter values were systematically tested (Supplementary Figure 20). The combination of parameter values that achieved the best performance were:
- `--tumor-mtc fdr`
- `--tumor-mtc-alpha 1`

VarScan2:

Tumor-only mode, default parameters:

```
samtools mpileup -f FASTA \
	TUMOR_BAM | path/to/varscan mpileup2cns \
		--output-vcf \
		--variants > NAME.vcf
```

VarScan2 needs a mpileup file (a text-based format file that summarizes base calls of aligned reads) as input, which SAMtools (Li et al., 2009) can build when using the `mpileup` command. Then, the VarScan2 `mpileup2cns` command is run to perform variant calling.

Tumor-normal paired mode, default parameters:

```
samtools mpileup -f FASTA \
        TUMOR_BAM NORMAL_BAM | path/to/varscan somatic \
                OUTPUT_NAME \
                --mpileup 1 \
                --output-vcf
```

In tumor-normal paired mode, a single mpileup is generated using both the tumor and the normal BAM files from the sample. Then, the `-mpileup 1` command from VarScan2 ensures a single BAM file is processed as input.

The limit of detection is removed by adding the following parameters:

- `--min-var-freq 0.0 (for varscan) [default: 0.01]:` minimum variant allele frequency threshold
- `-d 0 (for mpileup) [default: 8000]:` maximum reads read per input file

To detect the best set of parameters for **high-sensitivity settings**, we tested the following parameters on the training set containing only SNVs spiked-in:

- For tumor-only mode:
    - `--p-value [default: 99e-02]:` p-value threshold for calling variants
    - `--min-avg-qual [default: 15]:` minimum base quality at a position to count a read

- For tumor-normal paired mode:
    - `--p-value [default: 0.99]:` p-value threshold to call a heterozygote
    - `--somatic-p-value [default: 0.05]:` p-value threshold to call a somatic site

We lastly utilized the following combination of parameter values for **high-sensitivity settings**, after the benchmarking results (Supplementary Figure 21):

- For tumor-only mode:
    - `--p-value 0.1`
    - `--min-avg-qual 5`

- For tumor-normal paired mode:
    - `--p-value 0.1`
    - `--somatic-p-value 0.1`

Freebayes:

Tumor only mode, default parameters:

```
freebayes \
      -f FASTA \
      -b TUMOR_BAM
```

Tumor-normal paired mode, default parameters:

```
freebayes \
      -f FASTA \
      -b TUMOR_BAM \
      -b NORMAL_BAM
```

To remove the **limit of detection** we set the following parameter:

- `--min-alternate-fraction 0.01`

Further increasing the limit of detection was not possible, due to the high computational time required from the algorithm in such settings.

Strelka2:

Tumor-normal paired mode, default parameters:

```
path/to/strelka/bin/configureStrelkaSomaticWorkflow.py \\
      --tumorBam TUMOR_BAM \
      --normalBam NORMAL_BAM \
      --referenceFasta FASTA \
      --runDir ANALYSIS_DIR_PATH

ANALYSIS_DIR_PATH/runWorkflow.py -m local -j CPUS
```

`where`

- `ANALYSIS_DIR_PATH` is the folder, generated by Strelka2 itself, in which the analysis is run
- `-j CPUS` is the number of threads to be used

The following parameters were benchmarked in order to detect the best values for the **high-sensitivity settings** (values tested are reported):

- `depthFilterMultiple = [3.0, 100.0]`
- `snvMaxFilteredBasecallFrac = [1.0, 0.01]`
- `snvMaxSpanningDeletionFrac = [0.75, 0.01]`
- `indelMaxWindowFilteredBasecallFrac = [0.03, 3]`
- `minTier1Mapq = [10, 30]`
- `ssnvQuality_LowerBound = [1, 30]`
- `sindelQuality_LowerBound = [1, 60]`

S1.5. Variant calling metrics for benchmarking

Variant calling performances are evaluated using an in-house developed script (*performance_evaluation.py*) provided at https://github.com/DIncalciLab/dincalcilab-lowfrac-variant-benchmark It is written in Python programming language (version 3.9) and built around the *cyvcf2* package (Pedersen and Quinlan, 2017), a Cython (https://cython.org/) wrapper for fast parsing of VCF files, which allows the easy extraction of the variants from VCF files.

Called variants extracted are compared with those successfully spiked-in and four metrics are calculated: True Positive Rate (TPR), Positive Predictive Value (PPV),False Discovery Rate (FDR) and F1 score.

The **True Positive Rate** (or Sensitivity, or Recall) is calculated as:

$$TPR = \frac{TP}{TP + FN}$$

where:

- True Positive (TP) is the number of spiked-in somatic variants (SNVs or INDELs) correctly called
- False Negative (FN) is the number of spiked-in somatic variants not called

The **Positive Predictive Value** (or Precision) is calculated as:

$$PPV = \frac{TP}{TP + FP}$$

where:

- False Positive (FP) is the number of called somatic variants that were not spiked-in, thus are incorrectly called. Called pseudo-germline variants randomly inserted in the initial artificial normal samples are not considered as false positives, and thus removed from the calculation.

The **False Discovery Rate** is calculated as:

$$FDR = \frac{FP}{FP + TP} = 1 - PPV$$

The **F1 score** is calculated as:

$$F1score = 2\frac{PPV \cdot TPR}{PPV + TPR} = \frac{2TP}{2TP + FP + FN}$$

S1.6. BAM files downsampling

The random selection for reads downsampling was performed using the sambamba (Tarasov et al., 2015) tool. Specifically, for each percentage range (i.e., 2-8% and 20-80%), we used the `subsample` command in sambamba to randomly select the desired number of reads. The command takes into account the read group information, which allows for subsampling at the individual sample level. In particular, for each sample and each downsample step, we ran the following command:

```
sambamba
      view
      -s FRACTION
      --subsampling-seed 123
      -f bam
      -o OUTPUT_BAM
      INPUT_BAM
```

where:

- `-s [default: .01]:` fraction of reads from the original BAM to be retained
- `--subsampling-seed:` seed for subsampling
- `-f:` output format
- `-o:` output file name

S2. Supplementary Results

S2.1 Spike-in of artificial variants

We tested the reliability of the spike-in of artificial somatic variants by comparing the number of generated loci versus their actual number spiked in the data, i.e., the spike-in success rate. Performances of SNVs and INDELs were evaluated separately.

The SNV spike-in success rate for each sample is reported in Supplementary Table 1. There was no significant difference (Kolmogorov-Smirnov test, p-value = 0.99) in the VAF distribution of the generated variants versus the VAF distribution of the actual spiked-in variants, for both the entire artificial sample cohort (Supplementary Figure 1) and at the individual sample level (Supplementary Figure 2).

Supplementary Table 1. Success rate of spiked-in somatic SNVs. Success rate represents the ratio between the actual number of simulated random variants successfully inserted into the normal data and their generated number.

Sample	Simulated	Spiked-in	Success rate
SM_snv_100_01	100	100	1.00
SM_snv_100_02	100	100	1.00
SM_snv_100_03	100	100	1.00
SM_snv_100_04	100	100	1.00
SM_snv_100_05	100	99	0.99
SM_snv_100_06	100	100	1.00
SM_snv_100_07	100	100	1.00
SM_snv_100_08	100	99	0.99
SM_snv_100_09	100	99	0.99
SM_snv_100_10	100	100	1.00

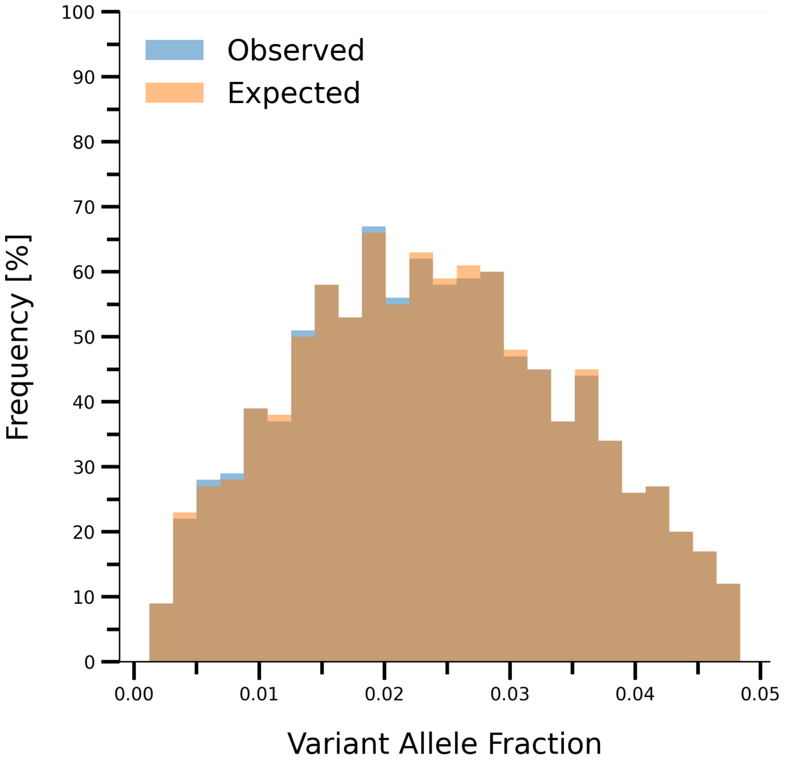

Supplementary Figure 1. Variant Allele Fraction distribution of all the expected (generated, to be spiked-in) and observed (after spiking-in) SNVs.

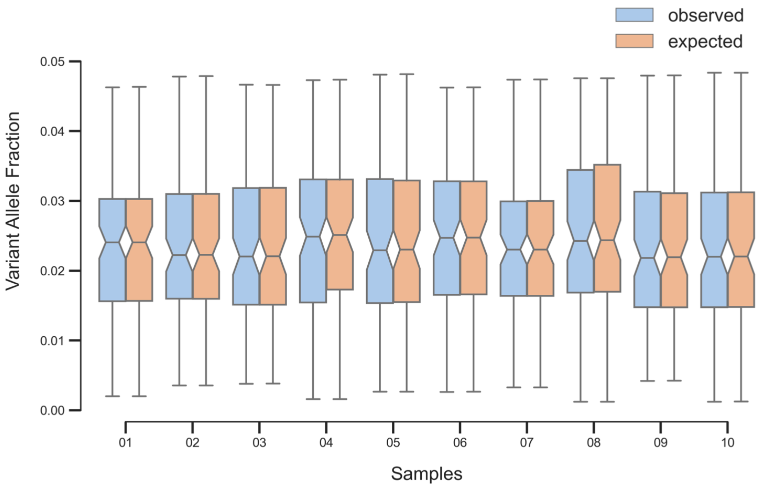

Supplementary Figure 2. Variant Allele Fraction distribution of expected (generated, to be spiked-in) and observed (after spiking-in) SNVs for each sample.

The spike-in success rates for both INDELs (maximum 90 bp long) and short INDELs (maximum 3 bp long) are reported in Supplementary Tables 2 and 3, respectively. As for SNVs, the VAF distributions of the INDELs were not significantly different at both the entire cohort (Kolmogorov-Smirnov test, p-value = 0.16 and p-value = 0.91 for INDELs and short INDELs, respectively) and single sample levels (Kolmogorov-Smirnov test, p-value > 0.05 for both INDELs and short INDELs) (Supplementary Figures 3 to 6).

Supplementary Table 2. Success rate of spiked-in somatic INDELs (max length 90 bp). Success rate represents the ratio between the actual number of simulated random variants successfully inserted into the normal data and their generated number.

Sample	Simulated	Spiked-in	Success rate
SM_indel_100_01	100	100	1.00
SM_indel_100_02	100	100	1.00
SM_indel_100_03	100	100	1.00
SM_indel_100_04	100	100	1.00
SM_indel_100_05	100	100	1.00
SM_indel_100_06	100	100	1.00
SM_indel_100_07	100	100	1.00
SM_indel_100_08	100	100	1.00
SM_indel_100_09	100	100	1.00
SM_indel_100_10	100	100	1.00

Supplementary Table 3. Success rate of spiked-in somatic short INDELs (max length 3 bp). Success rate represents the ratio between the actual number of simulated random variants successfully inserted into the normal data and their generated number.

Sample	Simulated	Spiked-in	Success rate
SM_indel3_100_01	100	100	1.00
SM_indel3_100_02	100	100	1.00
SM_indel3_100_03	100	100	1.00
SM_indel3_100_04	100	100	1.00
SM_indel3_100_05	100	100	1.00
SM_indel3_100_06	100	100	1.00
SM_indel3_100_07	100	100	1.00
SM_indel3_100_08	100	100	1.00
SM_indel3_100_09	100	100	1.00
SM_indel3_100_10	100	100	1.00

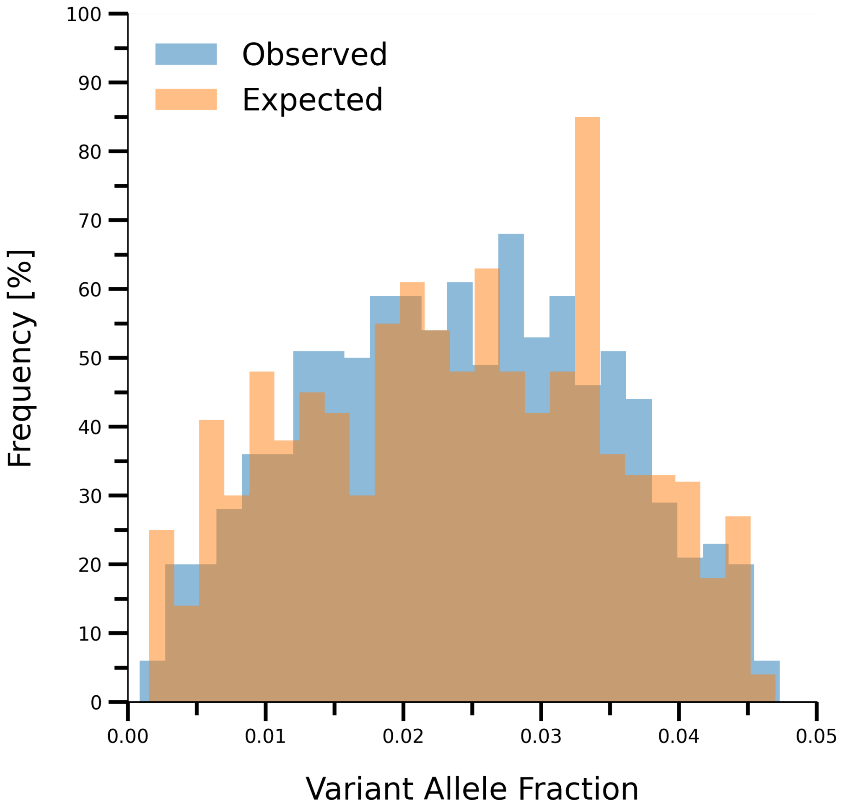

Supplementary Figure 3. Variant Allele Fraction distribution of expected (generated, to be spiked-in) and observed (after spiking-in) INDELs (max length 90 bp).

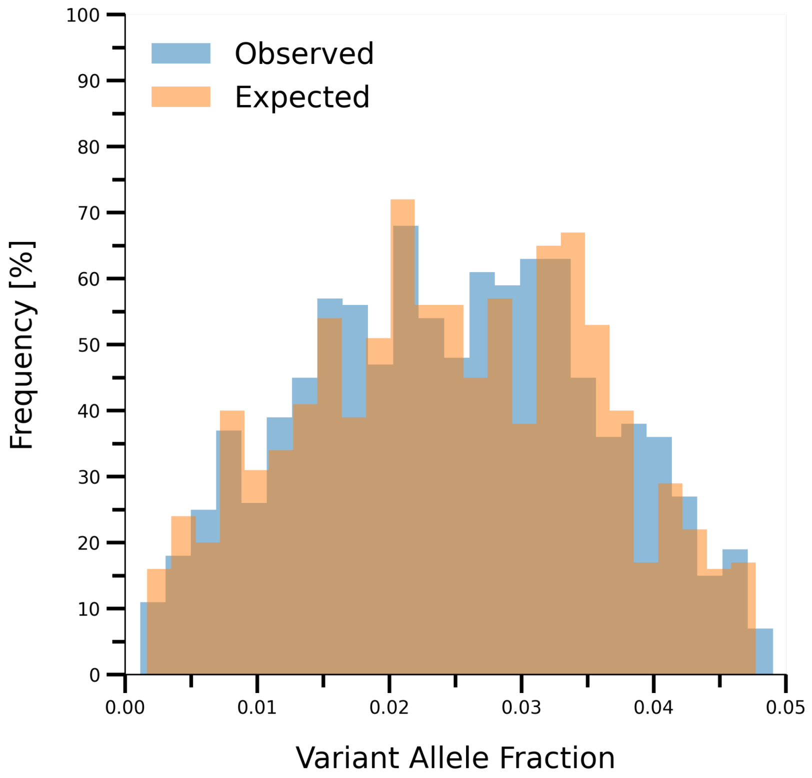

Supplementary Figure 4. Variant Allele Fraction distribution of expected (generated, to be spiked-in) and observed (after spiking-in) short INDELs (max length 3 bp).

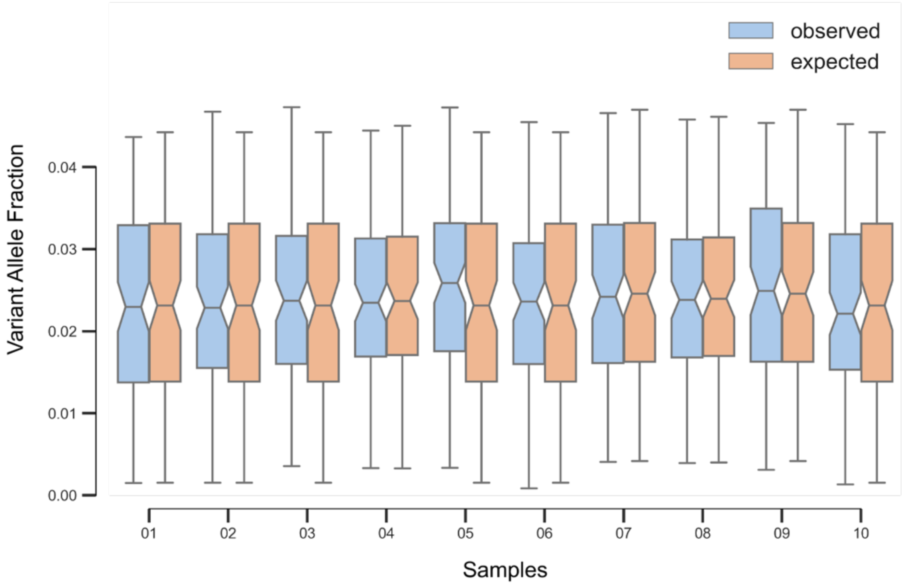

Supplementary Figure 5. Variant Allele Fraction distribution of expected (generated, to be spiked-in) and observed (after spiking-in) INDELs (max length 90 bp) for each sample.

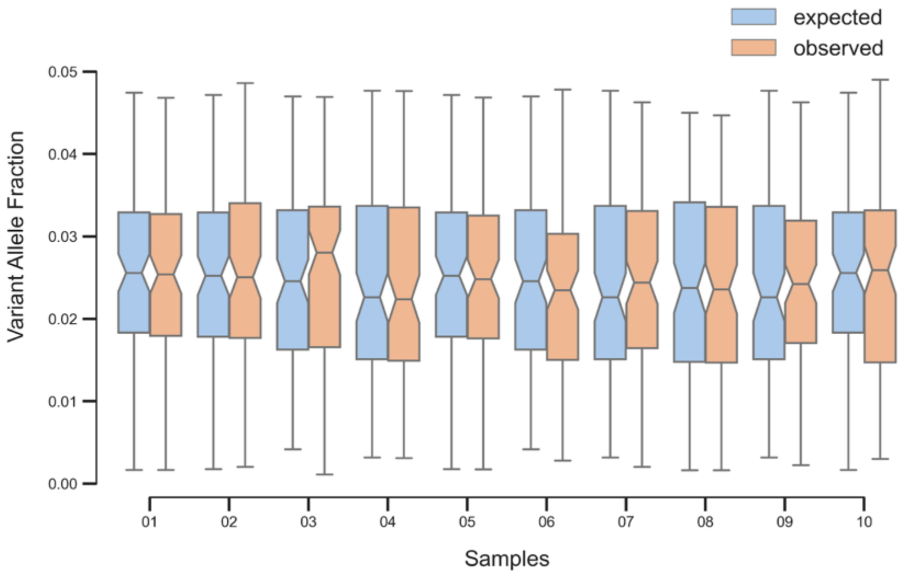

Supplementary Figure 6. Variant Allele Fraction distribution of expected (generated, to be spiked-in) and observed (after spiking-in) short INDELs (max length 3 bp) for each sample.

Moreover, to ensure the spike-in process does not alter the inserted INDELs, we calculated the size distributions of the generated and spiked-in INDELs; Supplementary Figure 7 reports them separately for deletions a) and insertions b), confirming that in both cases the length range of the observed spiked-in INDELs matches the range of the generated artificial loci.

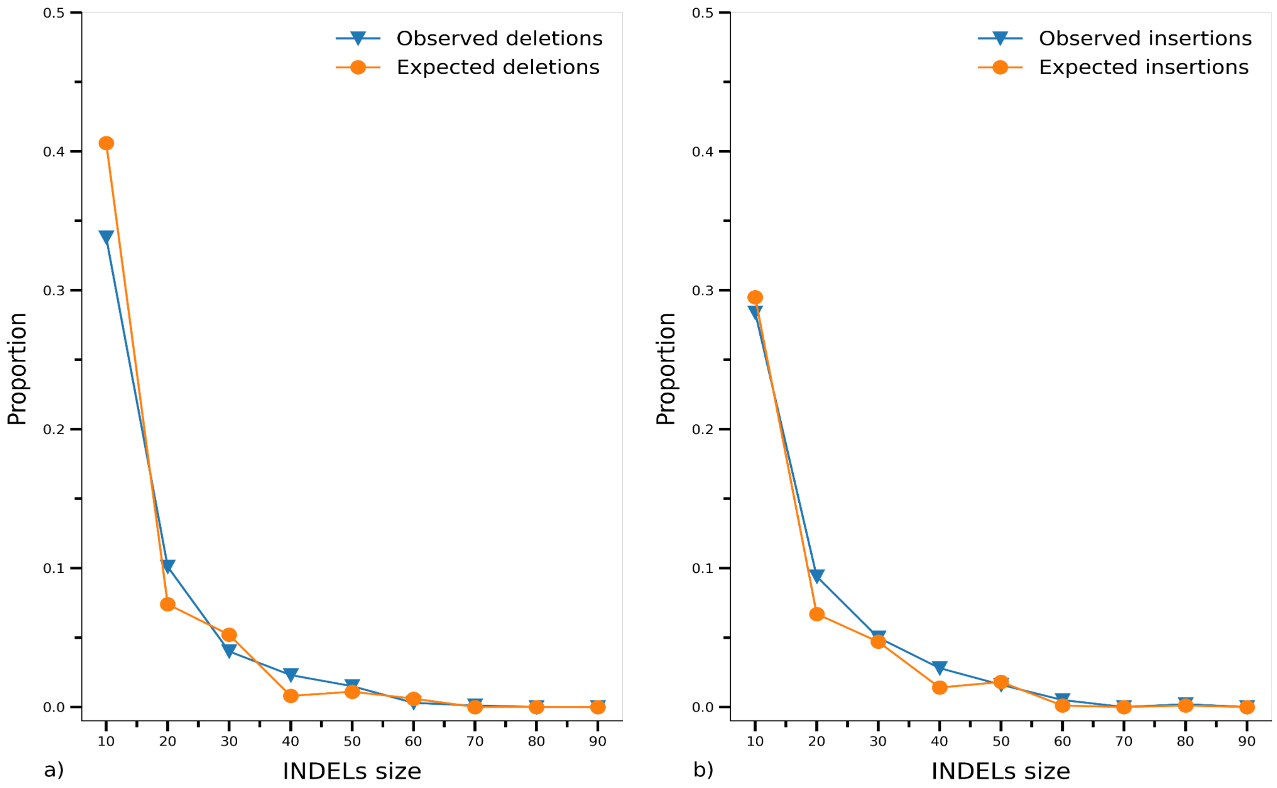

Supplementary Figure 7. Size distributions of expected (generated, to be spiked-in) and observed (after spiking-in) deletions a) and insertions b) (max length 90 bp). The proportions were computed, for each bin of 10 bp size, dividing the number of deletions or insertions with length within the specific bin lengths by the total number of INDEL events.

S2.2 Variant calling performance evaluation

Variant calling performances were evaluated through two distinct approaches, using three different types of artificial datasets and three different sets of parameter values. First, variant calling algorithms were tested using their parameter default values on a spiked-in SNVs only dataset, a spiked-in SNVs plus INDELs dataset, and a SNVs plus short INDELs dataset. These parameter values and datasets were first evaluated using a matched normal sample for each tumor dataset (tumor-normal paired mode). Then, using the same datasets, performances were assessed when running the calling algorithms removing the limit of detection (high-sensitivity settings) and using a tuned set of parameter values (Section S1.4). Lastly, the different datasets and sets of parameter values were tested without the use of a matched normal sample (tumor-only mode).

S2.2.1 Performance evaluation in default mode

Supplementary Tables 4-7 and Supplementary Figures 8 and 9 show the performance evaluation of the four variant callers (VarDict, Mutect2, LoFreq and Strelka2) used for the first benchmark, executed with their parameter default values and using the tumor-normal paired mode. Supplementary Table 8-11 and Supplementary Figures 10 and 11 show the performance of the variant callers under the same conditions, but using the tumor-only mode. FreeBayes and Varscan2 were not evaluated in this phase due to their limit of detection (minimum alternate allele fraction of 5%) on their parameter default values.

Tumor-normal paired mode

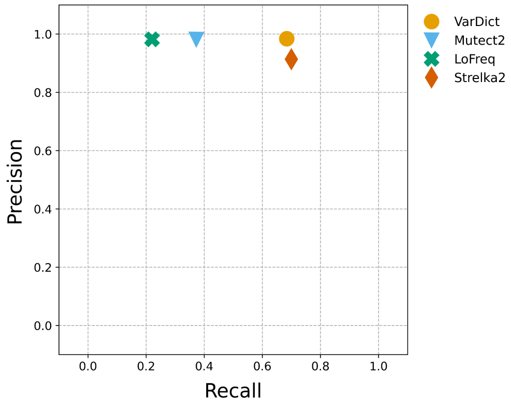

Supplementary Figure 8. Variant caller performance analysis with low-fraction spiked-in somatic SNVs, using tumor-normal paired mode and parameter default values. Plot depicts the recall (i.e., Sensitivity, or TPR) and precision (i.e., PPV) of VarDict, Mutect2, LoFreq and Strelka2 variant callers in tumor-normal paired mode with parameter default values on simulated data samples with spiked-in somatic SNVs only.

Supplementary Table 4. Performance of variant callers in calling SNVs using parameter default values in matched tumor-normal paired mode on SNVs spiked-in data.

Variant caller	VarDict	Mutect2	LoFreq	Strelka2
True Positive	682	372	220	698
False Positive	11	7	4	66
False Negative	315	625	777	299
True Positive Rate	0.684	0.373	0.220	0.700
Positive Predictive Value	0.984	0.981	0.982	0.913
False Discovery Rate	0.015	0.018	0.017	0.086
F1 score	0.807	0.530	0.362	0.793

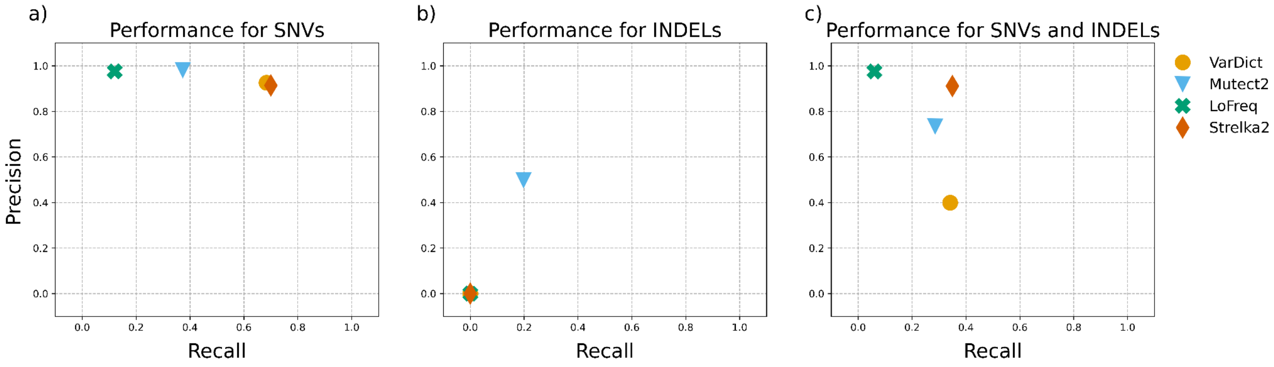

Supplementary Figure 9. Variant caller performance analysis with low-fraction spiked-in somatic SNVs and INDELs, using tumor-normal paired mode and parameter default values. Plots depict the recall (i.e., Sensitivity, or TPR) and precision (i.e., PPV) of VarDict, Mutect2, LoFreq and Strelka2 variant callers in tumor-normal paired mode with parameter default values on simulated data samples with spiked-in somatic SNVs and INDELs (max length 90 bp). Performances of the variant callers are reported for SNVs (a), INDELs (b), or both SNVs and INDELs (c).

Supplementary Table 5. Performance of variant callers in calling SNVs using parameter default values in matched tumor-normal paired mode on spiked-in data containing both SNVs and INDELs (max length 90 bp).

Variant caller	VarDict	Mutect2	LoFreq	Strelka2
True Positive	682	372	120	698
False Positive	55	7	3	66
False Negative	315	625	877	299
True Positive Rate	0.684	0.373	0.120	0.700
Positive Predictive Value	0.925	0.981	0.975	0.913
False Discovery Rate	0.074	0.018	0.024	0.086
F1 score	0.786	0.540	0.214	0.793

Supplementary Table 6. Performance of variant callers in calling INDELs (max length 90 bp) using parameter default values in matched tumor-normal paired mode on spiked-in data containing both SNVs and INDELs (max length 90 bp).

Variant caller	VarDict	Mutect2	LoFreq	Strelka2
True Positive	0	198	0	0
False Positive	972	199	0	2
False Negative	1,000	802	1,000	1,000
True Positive Rate	0.0	0.198	0.0	0.0
Positive Predictive Value	0.0	0.498	-	0.0
False Discovery Rate	1.0	0.501	-	1.0
F1 score	0.0	0.283	0.0	0.0

Supplementary Table 7. Performance of variant callers in calling both SNVs and INDELs (max length 90 bp) using parameter default values in matched tumor-normal paired mode on spiked-in data containing both SNVs and INDELs (max length 90 bp).

Variant caller	VarDict	Mutect2	LoFreq	Strelka2
True Positive	682	570	120	698
False Positive	1,027	206	3	68
False Negative	1,315	1,427	1,877	1,299
True Positive Rate	0.341	0.285	0.060	0.349
Positive Predictive Value	0.399	0.734	0.975	0.911
False Discovery Rate	0.600	0.265	0.024	0.088
F1 score	0.368	0.411	0.113	0.505

Tumor-only mode

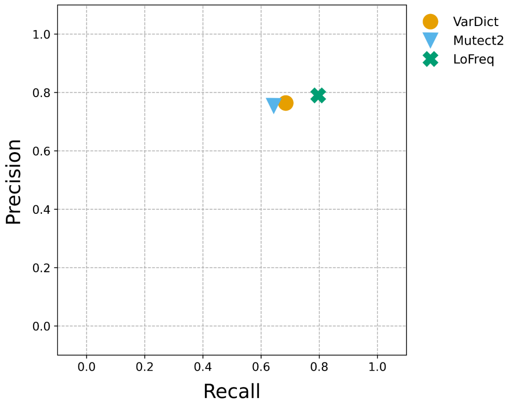

Supplementary Figure 10. Variant caller performance analysis with low-fraction spiked-in somatic SNVs, using tumor-only mode and parameter default values. Plot depicts the recall (i.e., Sensitivity, or TPR) and precision (i.e., PPV) of VarDict, Mutect2 and LoFreq variant callers in tumor-only mode with parameter default values on simulated data samples with spiked-in somatic SNVs.

Supplementary Table 8. Performance of variant callers in calling SNVs using parameter default values in tumor-only mode on SNVs spiked-in data.

Variant caller	VarDict	Mutect2	LoFreq
True Positive	683	642	794
False Positive	211	209	211
False Negative	314	355	203
True Positive Rate	0.685	0.643	0.796
Positive Predictive Value	0.763	0.754	0.790
False Discovery Rate	0.236	0.245	0.020
F1 score	0.722	0.695	0.793

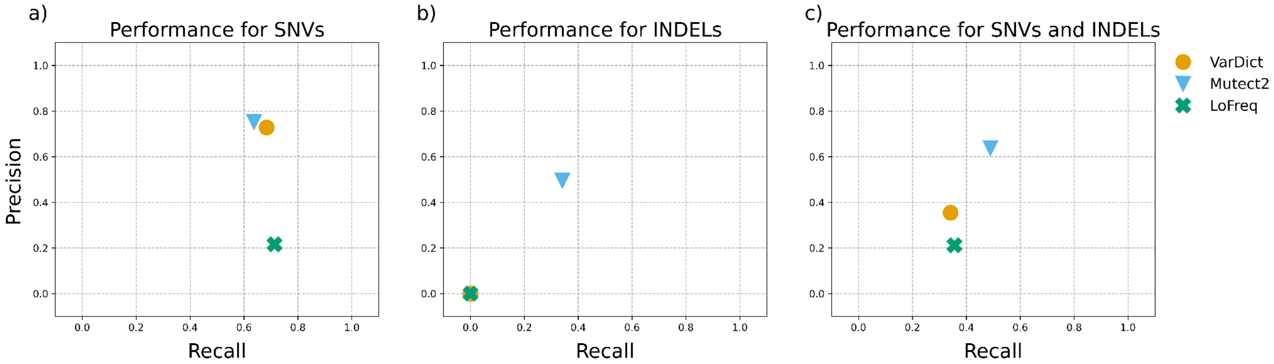

Supplementary Figure 11. Variant caller performance analysis with low-fraction spiked-in somatic SNVs and INDELs, using tumor-only mode and parameter default values. Plots depict the recall (i.e., Sensitivity, or TPR) and precision (i.e., PPV) of VarDict, Mutect2 and LoFreq variant callers in tumor-only mode with parameter default values on simulated data samples with spiked-in somatic SNVs and INDELs (max length 90 bp). Performances of the variant callers are reported for SNVs (a), INDELs (b), or both SNVs and INDELs (c).

Supplementary Table 9. Performance of variant callers in calling SNVs using parameter default values in tumor-only mode on spiked-in data containing both SNVs and INDELs (max length 90 bp).

Variant caller	VarDict	Mutect2	LoFreq
True Positive	682	635	711
False Positive	255	209	2,578
False Negative	315	362	286
True Positive Rate	0.684	0.636	0.713
Positive Predictive Value	0.727	0.752	0.216
False Discovery Rate	0.272	0.247	0.783
F1 score	0.705	0.690	0.331

Supplementary Table 10. Performance of variant callers in calling INDELs using parameter default values in tumor-only mode on spiked-in data containing both SNVs and INDELs (max length 90 bp).

Variant caller	VarDict	Mutect2	LoFreq
True Positive	0	341	0
False Positive	989	348	66
False Negative	1,000	659	1,000
True Positive Rate	0.0	0.341	0.0
Positive Predictive Value	0.0	0.494	0.0
False Discovery Rate	1.0	0.505	1.0
F1 score	0.0	0.404	0.0

Supplementary Table 11. Performance of variant callers in calling both SNVs and INDELs using parameter default values in tumor-only mode on spiked-in data containing both SNVs and INDELs (max length 90 bp).

Variant caller	VarDict	Mutect2	LoFreq
True Positive	682	977	711
False Positive	1,244	556	2,644
False Negative	1,315	1,020	1,286
True Positive Rate	0.341	0.489	0.356
Positive Predictive Value	0.354	0.637	0.211
False Discovery Rate	0.645	0.362	0.788
F1 score	0.348	0.554	0.266

S2.2.2 Performance evaluation without limit of detection

Performance evaluations of variant calling algorithms run when their limit of detection is removed are reported in Supplementary Tables 12-15 and Supplementary Figure 12 and 13 (for tumor-normal paired mode) and Supplementary Tables 16-19 and Supplementary Figures 14 and 15 (for tumor-only mode).

Tumor-normal paired mode, no limit of detection

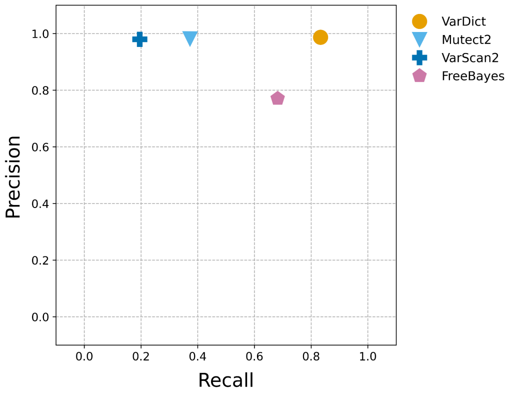

Supplementary Figure 12. Variant caller performance analysis with low-fraction spiked-in somatic SNVs, using tumor-normal paired mode and removing the limit of detection on variant fraction. Plot depicts the recall (i.e., Sensitivity, or TPR) and precision (i.e., PPV) of VarDict, Mutect2, VarScan2 and FreeBayes variant callers in tumor-normal paired mode with their limit of detection on the variant fraction removed, on simulated data samples with spiked-in somatic SNVs.

Supplementary Table 12. Performance of variant callers in calling SNVs in tumor-normal paired mode without their limit of detection, on spiked-in data containing only SNVs.

Variant caller	VarDict	Mutect2	VarScan2	FreeBayes
True Positive	831	372	195	680
False Positive	11	7	4	202
False Negative	166	625	802	317
True Positive Rate	0.833	0.373	0.195	0.682
Positive Predictive Value	0.986	0.981	0.979	0.770
False Discovery Rate	0.013	0.018	0.020	0.229
F1 score	0.904	0.541	0.326	0.724

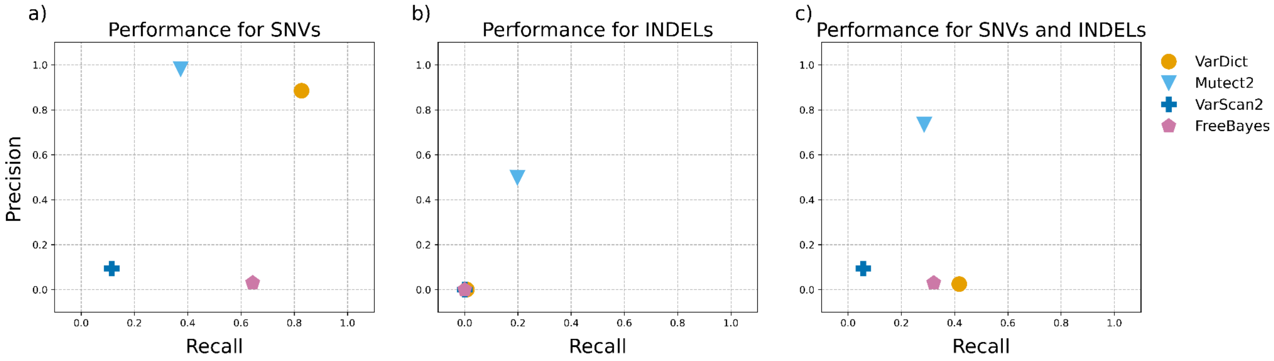

Supplementary Figure 13. Variant caller performance analysis with low-fraction spiked-in somatic SNVs and INDELs, using tumor-normal paired mode and removing the limit of detection on variant fraction. Plots depict the recall (i.e., Sensitivity, or TPR) and precision (i.e., PPV) of VarDict, Mutect2, VarScan2 and FreeBayes variant callers in tumor-normal paired mode with their limit of detection on the variant fraction removed, on simulated data samples with spiked-in somatic SNVs and INDELs. Performances of the variant callers are reported for SNVs (a), INDELs (b), or both SNVs and INDELs (c).

Supplementary Table 13. Performance of variant callers in calling SNVs in tumor-normal paired mode without their limit of detection, on spiked-in data containing both SNVs and INDELs (max length 90 bp).

Variant caller	VarDict	Mutect2	VarScan2	FreeBayes
True Positive	825	372	114	642
False Positive	107	7	1,102	20,395
False Negative	172	625	883	355
True Positive Rate	0.827	0.373	0.114	0.643
Positive Predictive Value	0.885	0.981	0.093	0.030
False Discovery Rate	0.114	0.018	0.906	0.969
F1 score	0.855	0.541	0.103	0.058

Supplementary Table 14. Performance of variant callers in calling INDELs (max length 90 bp) in tumor-normal paired mode without their limit of detection, on spiked-in data containing both SNVs and INDELs (max length 90 bp).

Variant caller	VarDict	Mutect2	VarScan2	FreeBayes
True Positive	8	198	0	0
False Positive	32,655	199	0	338
False Negative	992	802	1,000	1,000
True Positive Rate	0.008	0.198	0.0	0.0
Positive Predictive Value	0.0002	0.498	-	0.0
False Discovery Rate	0.999	0.501	-	1.0
F1 score	0.0004	0.283	0.0	0.0

Supplementary Table 15. Performance of variant callers in calling both SNVs and INDELs (max length 90 bp) in tumor-normal paired mode without their limit of detection, on spiked-in data containing both SNVs and INDELs (max length 90 bp).

Variant caller	VarDict	Mutect2	VarScan2	FreeBayes
True Positive	833	570	114	642
False Positive	32,762	206	1,102	20,733
False Negative	1,164	1,427	1,883	1,355
True Positive Rate	0.417	0.285	0.057	0.321
Positive Predictive Value	0.024	0.734	0.093	0.030
False Discovery Rate	0.975	0.265	0.906	0.969
F1 score	0.046	0.411	0.070	0.054

Tumor-only mode, no limit of detection

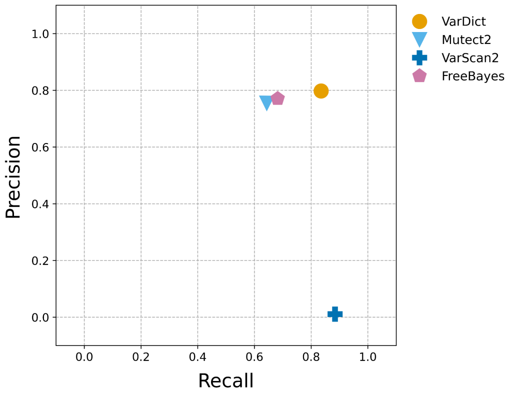

Supplementary Figure 14. Variant caller performance analysis with low-fraction spiked-in somatic SNVs, using tumor-only mode and removing the limit of detection on variant fraction. Plot depicts the recall (i.e., Sensitivity, or TPR) and precision (i.e., PPV) of VarDict, Mutect2, VarScan2 and FreeBayes variant callers in tumor-only mode with their limit of detection on the variant fraction removed, on simulated data samples with spiked-in somatic SNVs.

Supplementary Table 16. Performance of variant callers in calling SNVs in tumor-only mode without their limit of detection, on spiked-in data containing only SNVs.

Variant caller	VarDict	Mutect2	VarScan2	FreeBayes
True Positive	833	642	882	680
False Positive	211	209	79,902	202
False Negative	164	355	115	317
True Positive Rate	0.835	0.643	0.884	0.682
Positive Predictive Value	0.797	0.754	0.010	0.770
False Discovery Rate	0.202	0.245	0.989	0.229
F1 score	0.816	0.695	0.021	0.723

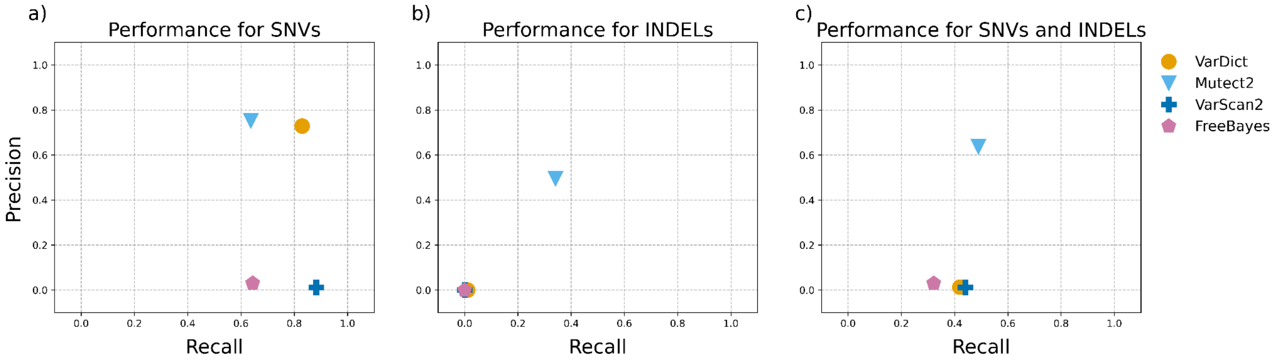

Supplementary Figure 15. Variant caller performance analysis with low-fraction spiked-in somatic SNVs and INDELs, using tumor-only mode and removing the limit of detection on variant fraction. Plots depict the recall (i.e., Sensitivity, or TPR) and precision (i.e., PPV) of VarDict, Mutect2, VarScan2 and FreeBayes variant callers in tumor-only mode with their limit of detection on the variant fraction removed, on simulated data samples with spiked-in somatic SNVs and INDELs. Performances of the variant callers are reported for SNVs (a), INDELs (b), or both SNVs and INDELs (c).

Supplementary Table 17. Performance of variant callers in calling SNVs in tumor-only mode without their limit of detection, on spiked-in data containing both SNVs and INDELs (max length 90 bp).

Variant caller	VarDict	Mutect2	VarScan2	FreeBayes
True Positive	827	635	880	642
False Positive	308	209	79,822	20,395
False Negative	170	362	117	355
True Positive Rate	0.829	0.636	0.882	0.643
Positive Predictive Value	0.728	0.752	0.010	0.030
False Discovery Rate	0.271	0.247	0.989	0.969
F1 score	0.776	0.609	0.021	0.058

Supplementary Table 18. Performance of variant callers in calling INDELs (max length 90 bp) in tumor-only mode without their limit of detection, on spiked-in data containing both SNVs and INDELs (max length 90 bp).

Variant caller	VarDict	Mutect2	VarScan2	FreeBayes
True Positive	11	341	0	0
False Positive	64,819	348	0	338
False Negative	989	659	1,000	1,000
True Positive Rate	0.011	0.341	0.0	0.0
Positive Predictive Value	0.0001	0.494	-	0.0
False Discovery Rate	0.999	0.505	-	1.0
F1 score	0.0003	0.404	0.0	0.0

Supplementary Table 19. Performance of variant callers in calling both SNVs and INDELs (max length 90 bp) in tumor-only mode without their limit of detection, on spiked-in data containing both SNVs and INDELs (max length 90 bp).

Variant caller	VarDict	Mutect2	VarScan2	FreeBayes
True Positive	838	977	880	642
False Positive	65,127	556	79,822	20,733
False Negative	1,159	1,020	1,117	1,355
True Positive Rate	0.419	0.489	0.440	0.321
Positive Predictive Value	0.013	0.637	0.010	0.030
False Discovery Rate	0.987	0.362	0.989	0.969
F1 score	0.024	0.553	0.021	0.055

S2.2.2 Performance evaluation in high-sensitivity settings

Benchmarks of parameter values, candidates to improve performance of variant callers on low-fraction variants, are reported in Supplementary Figure 16-20, for VarDict, Mutect2, LoFreq and VarScan2, respectively. FreeBayes parameter value benchmarks were not performed due to incorrect allelic fraction calling besides high computational time, while Strelka2 benchmarks are not reported as varying parameter values did not affect its outcomes. Performances using the tuned set of parameters (high-sensitivity settings) are reported in Supplementary Tables 20-27 for the training set, and in Supplementary Tables 28-35 for the test set.

VarDict candidate parameter values benchmark

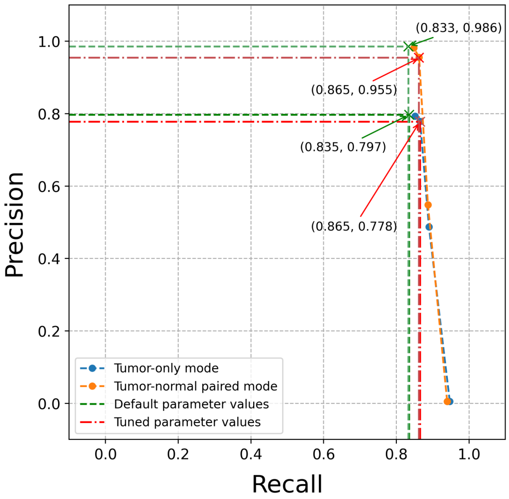

Supplementary Figure 16. Parameter benchmark for VarDict low-fraction variant calling tuning, on the training set containing only SNVs spiked-in. Plot depicts recall (i.e., Sensitivity, or TPR) and precision (i.e., PPV) of VarDict, when the candidate parameter values for low-fraction tuning are tested systematically on the training set, containing 699 SNVs spiked-in, for both tumor-normal paired mode and tumor-only mode. Green lines show the software performance when run using parameter default values but without the limit of detection, while red lines show the performance using the tuned parameter values.

Mutect2 candidate parameter values benchmark

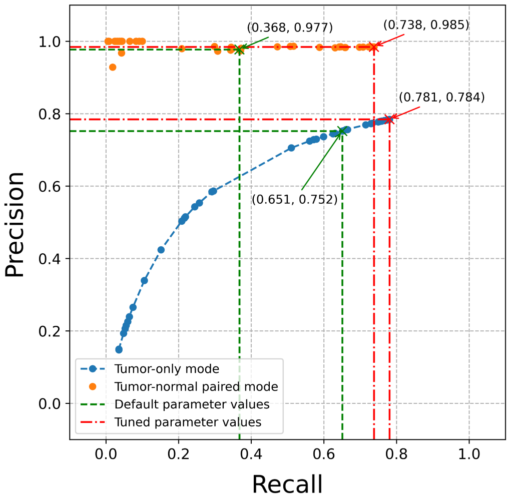

Supplementary Figure 17. Parameter benchmark for Mutect2 low-fraction variant calling tuning, on the training set containing only SNVs spiked-in. Plot depicts recall (i.e., Sensitivity, or TPR) and precision (i.e., PPV) of Mutect2, when the candidate parameters values for low-fraction variant calling tuning are tested systematically on the training set, containing 699 SNVs spiked-in, for both tumor-normal paired mode and tumor-only mode. Green lines show the software performance when run using parameter default values but without the limit of detection, while red lines show the performance using the tuned parameter values.

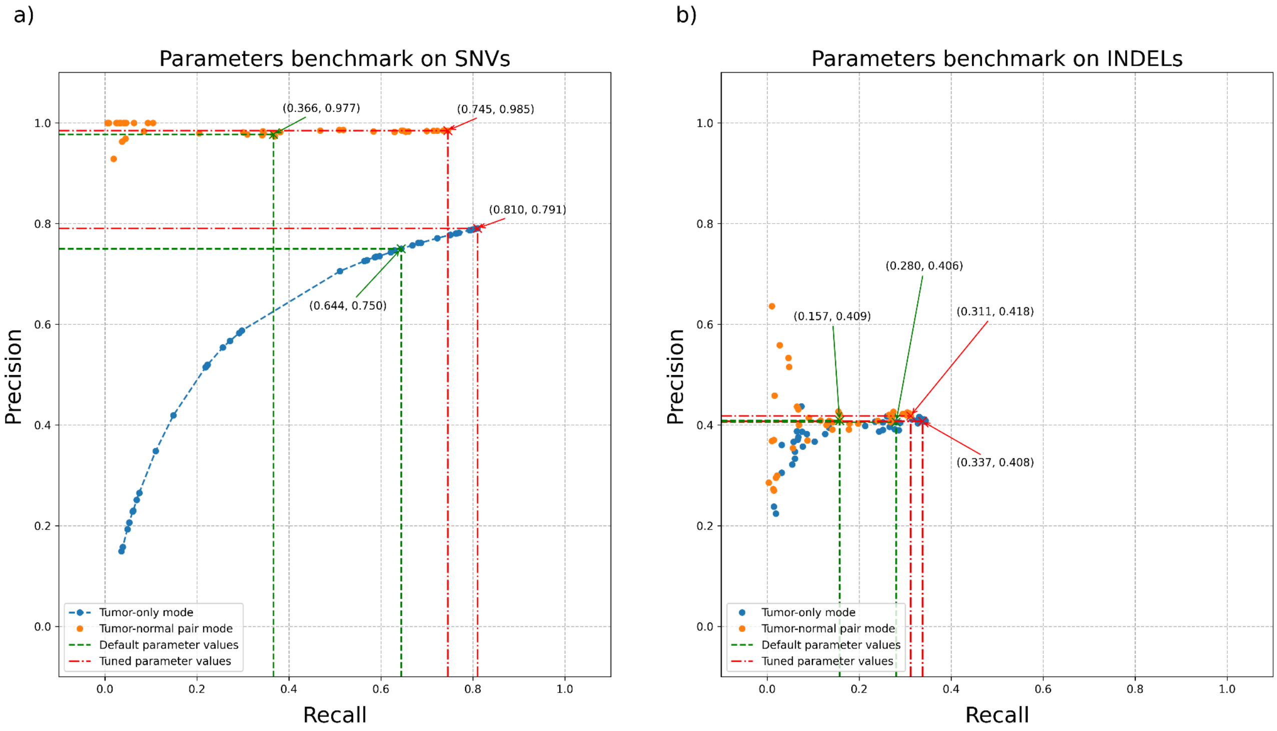

Supplementary Figure 18. Parameter benchmark for Mutect2 low-fraction variant calling tuning, on the training set containing SNVs and INDELs (max length 90 bp) spiked-in. Plots depict recall (i.e., Sensitivity, or TPR) and precision (i.e., PPV) of Mutect2, when the candidate parameter values for low-fraction tuning are tested systematically on the training set, containing 699 SNVs and 700 INDELs (max length 90 bp) spiked-in, for both tumor-normal paired mode and tumor-only mode. Green lines show the software performance when run using parameter default values but without the limit of detection, while red lines show the performance using the tuned parameter values.

LoFreq candidate parameter values benchmark

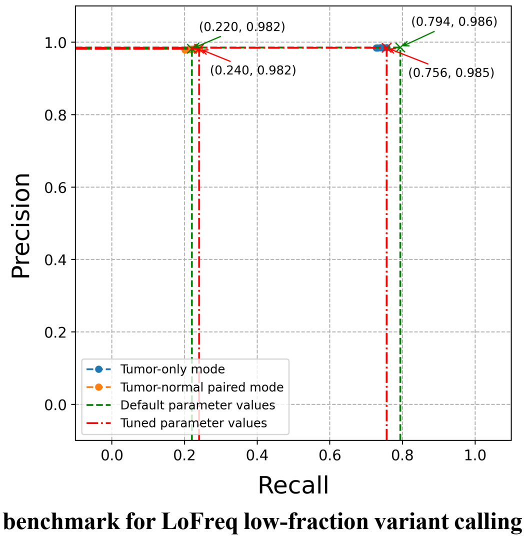

Supplementary Figure 19. Parameter benchmark for LoFreq low-fraction variant calling tuning, on the training set containing only SNVs spiked-in. Plot depicts recall (i.e., Sensitivity, or TPR) and precision (i.e., PPV) of LoFreq, when the candidate parameter values for low-fraction tuning are tested systematically on the training set, containing 699 SNVs spiked-in, for both tumor-normal paired mode and tumor-only mode. Green lines show the software performance when run using parameter default values but without the limit of detection, while red lines show the performance using the tuned parameter values.

VarScan2 candidate parameter values benchmark

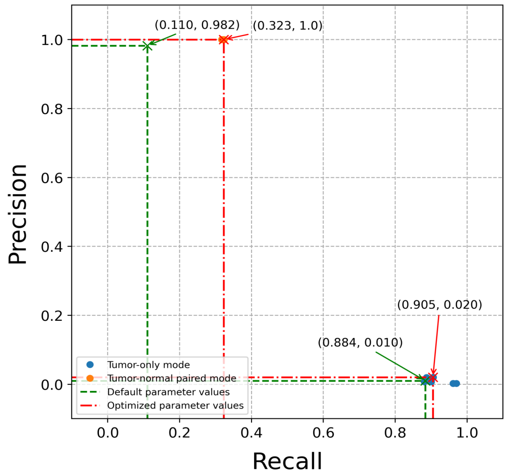

Supplementary Figure 20. Parameter benchmark for VarScan2 low-fraction variant calling tuning, on the training set containing only SNVs spiked-in. Plot depicts recall (i.e., Sensitivity, or TPR) and precision (i.e., PPV) of VarScan2, when the candidate parameter values for low-fraction tuning are tested systematically on the training set, containing 699 SNVs spiked-in, for both tumor-normal paired mode and tumor-only mode. Green lines show the software performance when run using parameter default values but without the limit of detection, while red lines show the performance using the tuned parameter values.

Tumor-normal paired mode, high-sensitivity settings, training set

Supplementary Table 20. Performance of variant callers in calling SNVs in tumor-normal paired mode using the tuned parameters, on the training set data containing only SNVs.

Variant caller	VarDict	Mutect2	LoFreq	VarScan2
True Positive	603	516	168	226
False Positive	28	8	3	0
False Negative	96	183	531	473
True Positive Rate	0.862	0.738	0.240	0.323
Positive Predictive Value	0.955	0.984	0.982	1.0
False Discovery Rate	0.044	0.015	0.017	0.0
F1 score	0.907	0.844	0.386	0.484

Supplementary Table 21. Performance of variant callers in calling SNVs in tumor-normal paired mode using the tuned parameters, on the training set data containing both SNVs and INDELs (max length 90 bp).

Variant caller	Mutect2
True Positive	521
False Positive	8
False Negative	178
True Positive Rate	0.745
Positive Predictive Value	0.984
False Discovery Rate	0.015
F1 score	0.849

Supplementary Table 22. Performance of variant callers in calling INDELs in tumor-normal paired mode using the tuned parameters, on the training set data containing both SNVs and INDELs (max length 90 bp).

Variant caller	Mutect2
True Positive	218
False Positive	303
False Negative	482
True Positive Rate	0.311
Positive Predictive Value	0.418
False Discovery Rate	0.581
F1 score	0.357

Supplementary Table 23. Performance of variant callers in calling both SNVs and INDELs in tumor-normal paired mode using the tuned parameters, on the training set data containing both SNVs and INDELs (max length 90 bp).

Variant caller	Mutect2
True Positive	739
False Positive	311
False Negative	660
True Positive Rate	0.528
Positive Predictive Value	0.703
False Discovery Rate	0.297
F1 score	0.604

Tumor-only mode, high-sensitivity settings, training set

Supplementary Table 24. Performance of variant callers in calling SNVs in tumor-only mode using the tuned parameters, on the training set data containing only SNVs.

Variant caller	VarDict	Mutect2	LoFreq	VarScan2
True Positive	833	546	529	633
False Positive	211	150	8	30,938
False Negative	164	153	170	66
True Positive Rate	0.835	0.781	0.756	0.905
Positive Predictive Value	0.797	0.784	0.986	0.020
False Discovery Rate	0.202	0.215	0.014	0.979
F1 score	0.816	0.783	0.856	0.392

Supplementary Table 25. Performance of variant callers in calling SNVs in tumor-only mode using the tuned parameters, on the training set data containing both SNVs and INDELs (max length 90 bp).

Variant caller	Mutect2
True Positive	566
False Positive	150
False Negative	133
True Positive Rate	0.809
Positive Predictive Value	0.790
False Discovery Rate	0.209
F1 score	0.800

Supplementary Table 26. Performance of variant callers in calling INDELs in tumor-only mode using the tuned parameters, on the training set data containing both SNVs and INDELs (max length 90 bp).

Variant caller	Mutect2
True Positive	236
False Positive	343
False Negative	464
True Positive Rate	0.337
Positive Predictive Value	0.407
False Discovery Rate	0.592
F1 score	0.369

Supplementary Table 27. Performance of variant callers in calling both SNVs and INDELs in tumor-only mode using the tuned parameters, on the training set data containing both SNVs and INDELs (max length 90 bp).

Variant caller	Mutect2
True Positive	802
False Positive	493
False Negative	597
True Positive Rate	0.573
Positive Predictive Value	0.619
False Discovery Rate	0.381
F1 score	0.595

Tumor-normal paired mode, high-sensitivity settings, test set

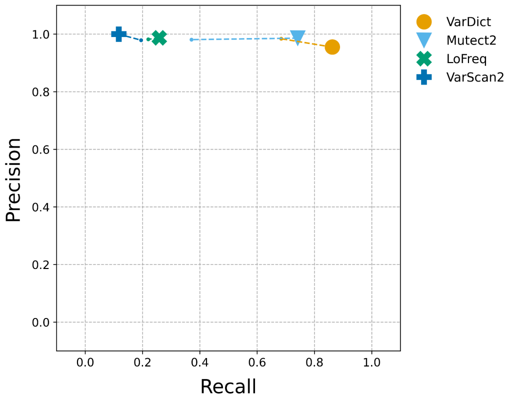

Supplementary Figure 21. Variant caller performance analysis with low-fraction spiked-in somatic SNVs, using tumor-normal paired mode and the tuned set of parameters. Plot depicts recall (i.e., Sensitivity, or TPR) and precision (i.e., PPV) of VarDict, Mutect2, LoFreq and VarScan2 variant callers in tumor-normal paired mode and using the tuned set of parameter values for each caller, on the test set with spiked-in somatic SNVs only. Dotted lines show the variation from the performance with parameter default values (for VarScan2 with the limit of detection removed).

Supplementary Table 28. Performance of variant callers in calling SNVs in tumor-normal paired mode using the tuned set of parameters, on the test set data containing only SNVs.

Variant caller	VarDict	Mutect2	VarScan2	LoFreq
True Positive	257	221	35	77
False Positive	12	3	0	1
False Negative	41	77	263	221
True Positive Rate	0.862	0.741	0.117	0.258
Positive Predictive Value	0.955	0.986	1.0	0.987
False Discovery Rate	0.044	0.013	0.0	0.012
F1 score	0.907	0.847	0.210	0.410

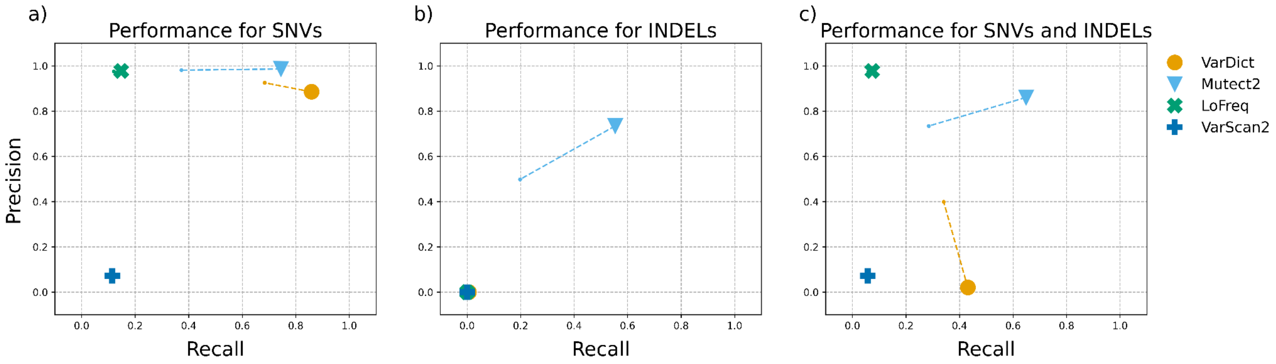

Supplementary Figure 22. Variant caller performance analysis with low-fraction spiked-in somatic SNVs and INDELs (max length 90 bp), using tumor-normal paired mode and the tuned set of parameters. Plots depict recall (i.e., Sensitivity, or TPR) and precision (i.e., PPV) of VarDict, Mutect2, LoFreq and VarScan2 variant callers in tumor-normal paired mode using the tuned set of parameter values for each caller, on the test set with spiked-in somatic SNVs and INDELs (max length 90 bp). Dotted lines show the variation from the performance with parameter default values (for VarScan2 with the limit of detection removed). Performances of variant callers are reported for SNVs (a), INDELs (b), or both SNVs and INDELs (c).

Supplementary Table 29. Performance of variant callers in calling SNVs in tumor-normal paired mode using the tuned set of parameters, on the test set data containing both SNVs and INDELs (max length 90 bp).

Variant caller	VarDict	Mutect2	VarScan2	LoFreq
True Positive	256	222	34	44
False Positive	33	3	437	1
False Negative	42	76	264	254
True Positive Rate	0.859	0.744	0.114	0.147
Positive Predictive Value	0.885	0.986	0.072	0.977
False Discovery Rate	0.114	0.013	0.927	0.022
F1 score	0.872	0.849	0.088	0.257

Supplementary Table 30. Performance of variant callers in calling INDELs in tumor-normal paired mode using the tuned set of parameters, on the test set data containing both SNVs and INDELs (max length 90 bp).

Variant caller	VarDict	Mutect2	VarScan2	LoFreq
True Positive	2	166	0	0
False Positive	12,237	60	0	0
False Negative	298	134	300	300
True Positive Rate	0.006	0.553	0.0	0.0
Positive Predictive Value	0.0001	0.734	-	-
False Discovery Rate	0.999	0.265	-	-
F1 score	0.0003	0.631	0.0	0.0

Supplementary Table 31. Performance of variant callers in calling both SNVs and INDELs in tumor-normal paired mode using the tuned set of parameters, on the test set data containing both SNVs and INDELs (max length 90 bp).

Variant caller	VarDict	Mutect2	VarScan2	LoFreq
True Positive	258	388	34	44
False Positive	12,270	63	437	1
False Negative	340	210	564	554
True Positive Rate	0.431	0.648	0.056	0.073
Positive Predictive Value	0.020	0.860	0.072	0.977
False Discovery Rate	0.979	0.139	0.927	0.022
F1 score	0.039	0.740	0.064	0.137

Tumor-only mode, high-sensitivity settings, test set

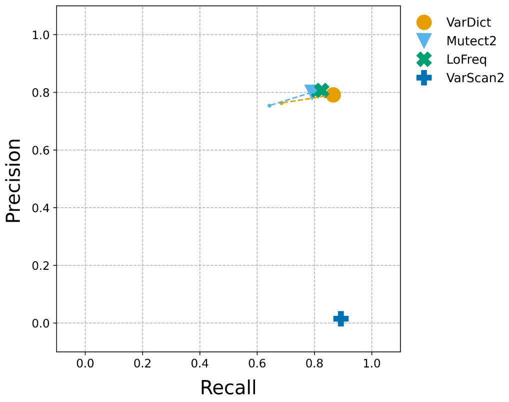

Supplementary Figure 23. Variant caller performance analysis with low-fraction spiked-in somatic SNVs, using tumor-only mode and the tuned set of parameters. Plot depicts recall (i.e., Sensitivity, or TPR) and precision (i.e., PPV) of VarDict, Mutect2, LoFreq and VarScan2 variant callers in tumor-only mode using the tuned set of parameter values for each caller, on the test set with spiked-in somatic SNVs only. Dotted lines show the variation from the performance with parameter default values (for VarScan2 with the limit of detection removed).

Supplementary Table 32. Performance of variant callers in calling SNVs in tumor-only mode using the tuned set of parameters, on the test set data containing only SNVs.

Variant caller	VarDict	Mutect2	VarScan2	LoFreq
True Positive	258	236	266	246
False Positive	68	59	17,393	59
False Negative	40	62	32	52
True Positive Rate	0.865	0.791	0.892	0.825
Positive Predictive Value	0.791	0.800	0.015	0.806
False Discovery Rate	0.208	0.200	0.984	0.193
F1 score	0.827	0.796	0.030	0.816

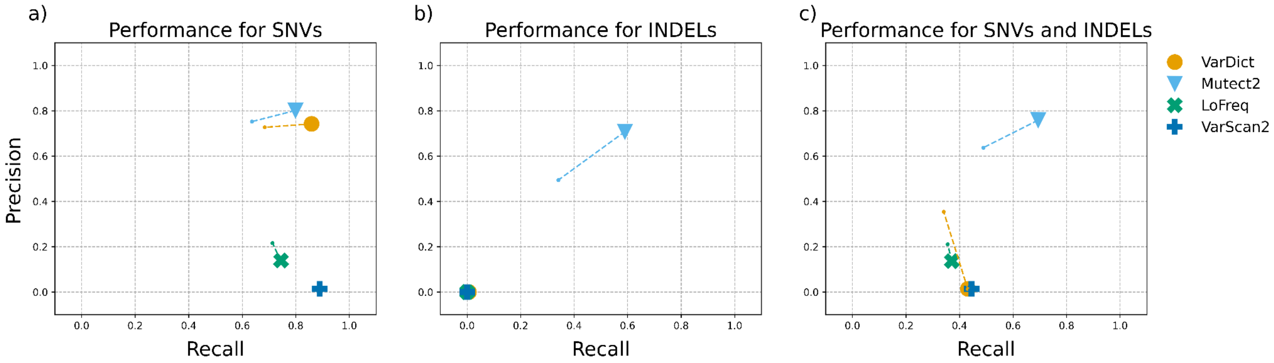

Supplementary Figure 24. Variant caller performance analysis with low-fraction spiked-in somatic SNVs and INDELs (max length 90 bp), using tumor-only mode and the tuned set of parameters. Plots depict recall (i.e., Sensitivity, or TPR) and precision (i.e., PPV) of VarDict, Mutect2, LoFreq and VarScan2 variant callers in tumor-only mode using the tuned set of parameter values for each caller, on the test set with spiked-in somatic SNVs and INDELs (max length 90 bp). Dotted lines show the variation from the performance with parameter default values (for VarScan2 with the limit of detection removed). Performances of variant callers are reported for SNVs (a), INDELs (b), or both SNVs and INDELs (c).

Supplementary Table 33. Performance of variant callers in calling SNVs in tumor-only mode using the tuned set of parameters, on the test set data containing SNVs and INDELs (max length 90 bp).

Variant caller	VarDict	Mutect2	VarScan2	LoFreq
True Positive	256	238	265	222
False Positive	89	59	18,570	1,376
False Negative	42	60	33	76
True Positive Rate	0.859	0.798	0.889	0.744
Positive Predictive Value	0.742	0.801	0.014	0.138
False Discovery Rate	0.257	0.198	0.985	0.861
F1 score	0.796	0.800	0.028	0.234

Supplementary Table 34. Performance of variant callers in calling INDELs in tumor-only mode using the tuned set of parameters, on the test set data containing SNVs and INDELs (max length 90 bp).

Variant caller	VarDict	Mutect2	VarScan2	LoFreq
True Positive	2	177	0	0
False Positive	18,097	73	0	28
False Negative	298	123	300	300
True Positive Rate	0.006	0.590	0.0	0.0
Positive Predictive Value	0.0001	0.708	-	0.0
False Discovery Rate	0.999	0.292	-	1.0
F1 score	0.0002	0.644	0.0	0.0

Supplementary Table 35. Performance of variant callers in calling SNVs and INDELs in tumor-only mode using the tuned set of parameters, on the test set data containing SNVs and INDELs (max length 90 bp).

Variant caller	VarDict	Mutect2	VarScan2	LoFreq
True Positive	258	415	266	222
False Positive	18,186	132	18,569	1,404
False Negative	340	183	332	376
True Positive Rate	0.431	0.693	0.444	0.371
Positive Predictive Value	0.013	0.758	0.014	0.136
False Discovery Rate	0.986	0.241	0.985	0.863
F1 score	0.027	0.725	0.027	0.200

S2.2.3 Performance evaluation on dataset containing SNVs and short INDELs (max length 3 bp)

Performance evaluations of variant calling algorithms run on the dataset containing SNVs and short INDELs (max length 3 bp) are reported in Supplementary Figure 21 and Supplementary Tables 36-38 for tumor-normal paired mode, and in Supplementary Figure 22 and Supplementary Table 39-41 for tumor-only mode. VarDict, Mutect2 and LoFreq were run using the tuned set of parameter values; FreeBayes was run with the limit of detection removed; Strelka2 was run using the parameter default values.

Tumor-normal paired mode

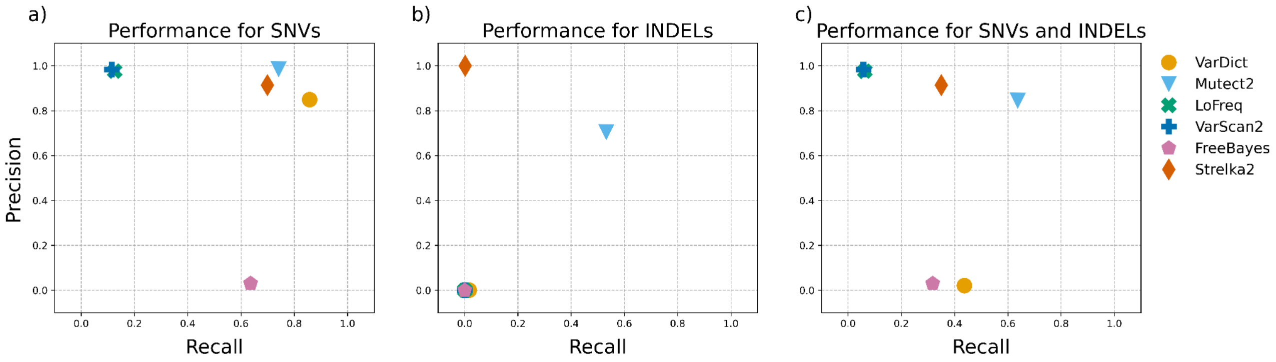

Supplementary Figure. 25. Variant caller performance analysis with low-fraction spiked-in somatic SNVs and short INDELs (max length 3 bp), using tumor-normal paired mode. Plots depict the recall (i.e., Sensitivity, or TPR) and precision (i.e., PPV) of VarDict, Mutect2, LoFreq, VarScan2, FreeBayes and Strelka2 variant callers in tumor-normal paired mode, on simulated data samples with spiked-in somatic SNVs and short INDELs (max length 3 bp). Performances of the variant callers are reported for SNVs (a), INDELs (b), or both SNVs and INDELs (c).

Supplementary Table 36. Performance of variant callers in calling SNVs in tumor-normal paired mode, on the dataset containing SNVs and short INDELs (max length 3 bp).

Variant caller	VarDict	Mutect2	LoFreq	VarScan2	FreeBayes	Strelka2
True Positive	855	739	125	115	634	697
False Positive	152	11	3	2	19,939	66
False Negative	142	258	872	882	363	300
True Positive Rate	0.857	0.741	0.125	0.115	0.635	0.699
Positive Predictive Value	0.849	0.985	0.976	0.982	0.030	0.913
False Discovery Rate	0.150	0.014	0.023	0.017	0.969	0.086
F1 score	0.853	0.846	0.222	0.206	0.059	0.792

Supplementary Table 37. Performance of variant callers in calling INDELs in tumor-normal paired mode , on the dataset containing SNVs and short INDELs (max length 3 bp).

Variant caller	VarDict	Mutect2	LoFreq	VarScan2	FreeBayes	Strelka2
True Positive	17	532	0	0	0	2
False Positive	41,406	222	0	0	395	0
False Negative	983	468	1000	1000	1000	998
True Positive Rate	0.017	0.532	0.0	0.0	0.0	0.002
Positive Predictive Value	0.0004	0.705	0.0	-	0.0	1
False Discovery Rate	0.999	0.294	0.0	-	1.0	0
F1 score	0.0008	0.607	0.0	0.0	0.0	0.004

Supplementary Table 38. Performance of variant callers in calling SNVs and INDELs in tumor-normal paired mode, on the dataset containing SNVs and short INDELs (max length 3 bp).

Variant caller	VarDict	Mutect2	LoFreq	VarScan2	FreeBayes	Strelka2
True Positive	872	1,272	125	115	634	699
False Positive	41,558	232	3	2	20,334	66
False Negative	1,125	725	1,872	1,882	1,363	1,298
True Positive Rate	0.436	0.636	0.062	0.057	0.317	0.350
Positive Predictive Value	0.020	0.845	0.976	0.982	0.030	0.913
False Discovery Rate	0.979	0.154	0.023	0.017	0.969	0.086
F1 score	0.039	0.727	0.118	0.109	0.055	0.506

Tumor-only mode

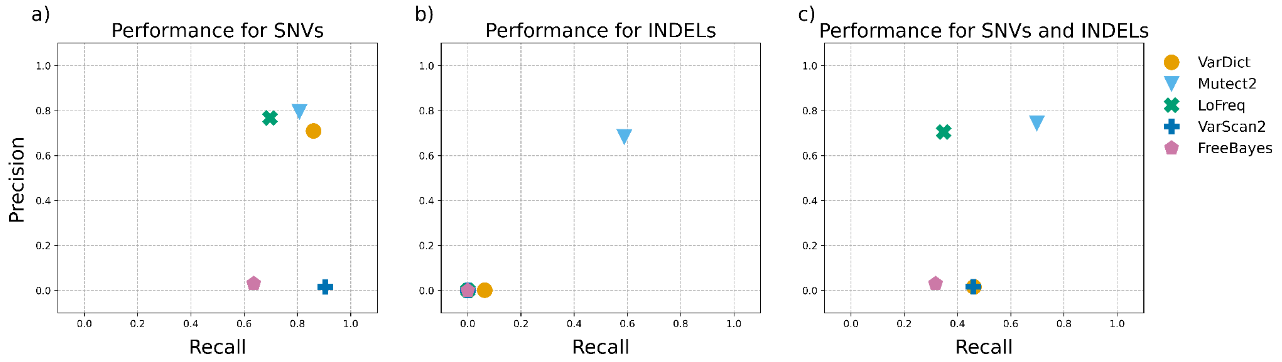

Supplementary Figure 26. Variant caller performance analysis with low-fraction spiked-in somatic SNVs and short INDELs (max length 3 bp), using tumor-only mode. Plots depict the recall (i.e., Sensitivity, or TPR) and precision (i.e., PPV) of VarDict, Mutect2, LoFreq, VarScan2 and FreeBayes variant callers in tumor-only mode, on simulated data samples with spiked-in somatic SNVs and short INDELs (max length 3 bp). Performances of the variant callers are reported for SNVs (a), INDELs (b), or both SNVs and INDELs (c).

Supplementary Table 39. Performance of variant callers in calling SNVs in tumor-only mode, on the dataset containing SNVs and short INDELs (max length 3 bp).

Variant caller	VarDict	Mutect2	LoFreq	VarScan2	FreeBayes
True Positive	858	805	695	902	634
False Positive	351	208	211	57,248	19,939
False Negative	139	192	302	95	363
True Positive Rate	0.860	0.807	0.697	0.904	0.635
Positive Predictive Value	0.709	0.794	0.767	0.015	0.030
False Discovery Rate	0.290	0.205	0.232	0.984	0.969
F1 score	0.778	0.800	0.730	0.030	0.059

Supplementary Table 40. Performance of variant callers in calling INDELs in tumor-only mode, on the dataset containing SNVs and short INDELs (max length 3 bp).

Variant caller	VarDict	Mutect2	LoFreq	VarScan2	FreeBayes
True Positive	64	588	0	0	0
False Positive	57,675	273	80	0	395
False Negative	936	412	1000	1000	1000
True Positive Rate	0.064	0.588	0.0	0.0	0.0
Positive Predictive Value	0.001	0.682	0.0	-	0.0
False Discovery Rate	0.998	0.317	1.0	-	1.0
F1 score	0.002	0.632	0.0	0.0	0.0

Supplementary Table 41. Performance of variant callers in calling SNVs and INDELs in tumor-only mode, on the dataset containing SNVs and short INDELs (max length 3 bp).

Variant caller	VarDict	Mutect2	LoFreq	VarScan2	FreeBayes
True Positive	922	1394	695	917	634
False Positive	58,026	480	291	57,233	20,334
False Negative	1,075	603	1,302	1,080	1,363
True Positive Rate	0.461	0.698	0.348	0.459	0.317
Positive Predictive Value	0.015	0.743	0.704	0.015	0.030
False Discovery Rate	0.984	0.256	0.295	0.984	0.969
F1 score	0.030	0.720	0.466	0.030	0.055

S2.2.4 Performance evaluation on downsampled samples

Performance evaluations of variant calling algorithms run on the downsampled datasets are reported in Supplementary Figures 23 and 24 for the datasets containing only SNVs, and in Supplementary Figures 25 and 26 for the datasets containing SNVs and INDELs. VarDict, Mutect2, LoFreq and VarScan2 were run using the tuned set of parameter values; FreeBayes was run with the limit of detection removed; Strelka2 was run using the parameter default values.

Performance evaluation on downsampled samples containing only SNVs

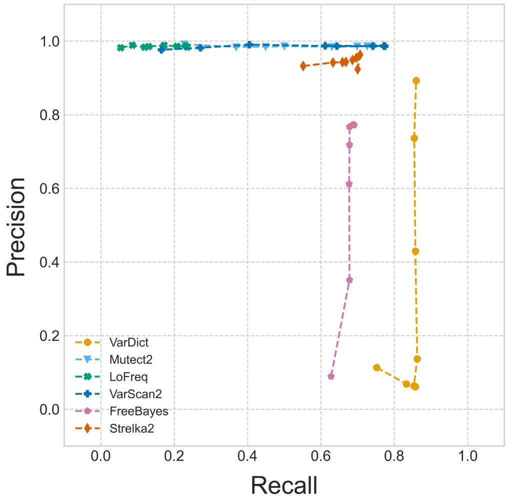

Supplementary Figure 27. Variant calling performance evaluation on downsampled samples containing only SNVs, in tumor-normal paired mode. Plot depicts recall (i.e., Sensitivity, or TPR) and precision (i.e., PPV) of VarDict, Mutect2, LoFreq, VarScan2, FreeBayes and Strelka2 in tumor-normal paired mode, on the datasets containing only SNVs. Each point on the plot corresponds to a specific read fraction percentage, representing the performance of the variant caller when tested on BAM files with read fractions ranging from 2% to 8% and 20% to 80%, with increments of 2% and 20%, respectively. The points are ordered from left to right according to increasing read fraction percentage, with the performance of each caller represented by a distinct color and symbol.

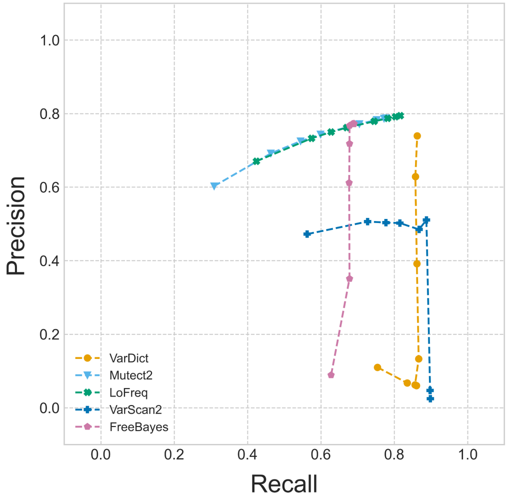

Supplementary Figure 28. Variant calling performance evaluation on downsampled samples containing only SNVs, in tumor-only mode. Plot depicts recall (i.e., Sensitivity, or TPR) and precision (i.e., PPV) of VarDict, Mutect2, LoFreq, VarScan2 and FreeBayes in tumor-only mode, on the datasets containing only SNVs. Each point on the plot corresponds to a specific read fraction percentage, representing the performance of the variant caller when tested on BAM files with read fractions ranging from 2% to 8% and 20% to 80%, with increments of 2% and 20%, respectively. The points are ordered from left to right according to increasing read fraction percentage, with the performance of each caller represented by a distinct color and symbol.

Performance evaluation on downsampled samples containing SNVs and INDELs

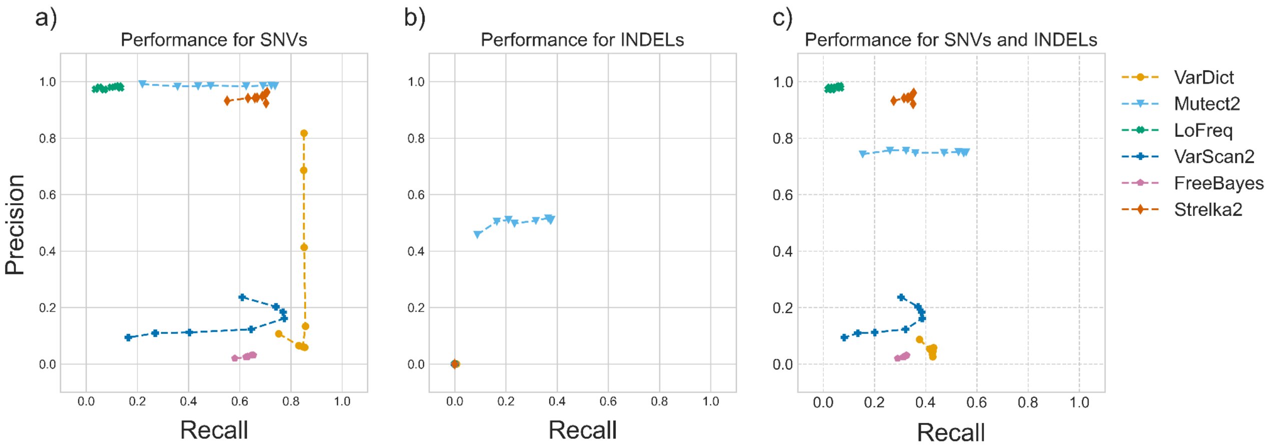

Supplementary Figure 29. Variant calling performance evaluation on downsampled samples containing SNVs and INDELs, in tumor-normal paired mode. Plots depict recall (i.e., Sensitivity, or TPR) and precision (i.e., PPV) of VarDict, Mutect2, LoFreq, VarScan2, FreeBayes and Strelka2 in tumor-normal paired mode, on the datasets containing SNVs and INDELs. Each point on the plot corresponds to a specific read fraction percentage, representing the performance of the variant caller when tested on BAM files with read fractions ranging from 2% to 8% and 20% to 80%, with increments of 2% and 20%, respectively. The points are ordered from left to right according to increasing read fraction percentage, with the performance of each caller represented by a distinct color and symbol. Performances of the variant callers are reported for SNVs (a), INDELs (b), or both SNVs and INDELs (c).

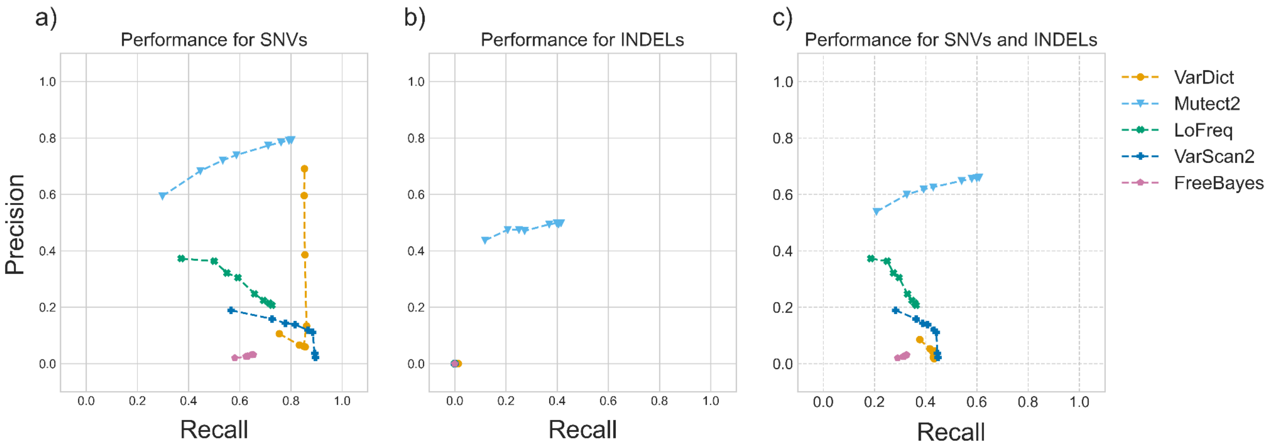

Supplementary Figure 30. Variant calling performance evaluation on downsampled samples containing SNVs and INDELs, in tumor-only mode. Plots depict recall (i.e., Sensitivity, or TPR) and precision (i.e., PPV) of VarDict, Mutect2, LoFreq, VarScan2 and FreeBayes in tumor-only mode, on the datasets containing SNVs and INDELs. Each point on the plot corresponds to a specific read fraction percentage, representing the performance of the variant caller when tested on BAM files with read fractions ranging from 2% to 8% and 20% to 80%, with increments of 2% and 20%, respectively. The points are ordered from left to right according to increasing read fraction percentage, with the performance of each caller represented by a distinct color and symbol. Performances of the variant callers are reported for SNVs (a), INDELs (b), or both SNVs and INDELs (c).

Performance evaluation on samples containing different numbers of SNVs

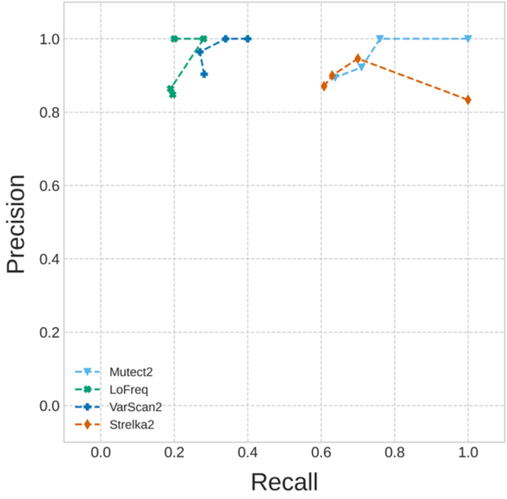

Supplementary Figure 31. Variant calling performance evaluation on downsampled samples containing different numbers of SNVs, in tumor-normal paired mode. Plot depicts recall (i.e., Sensitivity, or TPR) and precision (i.e., PPV) of Mutect2, LoFreq, VarScan2 and Strelka2, on the samples downsampled to 10,000X and containing different numbers of SNVs. Each point on the plot corresponds to a specific number of variants spiked in, representing the performance of the variant caller when tested using the high-sensitivity settings on downsampled BAM files with 200, 100, 50 and 10 SNVs inserted, respectively. The points are ordered from left to right according to decreasing SNVs inserted, with the performance of each caller represented by a distinct color and symbol.

Performance evaluation in comparison with DREAM challenge dataset

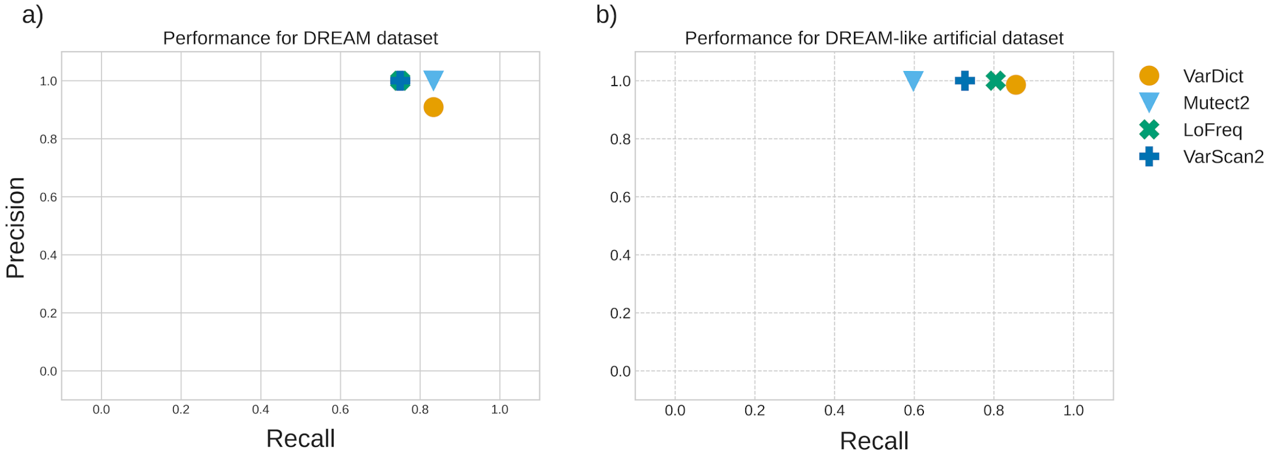

Supplementary Figure 32. Variant calling performance evaluation on the DREAM challenge dataset and 10 DREAM-like artificial samples, in tumor-normal paired mode. Plots depict recall (i.e., Sensitivity, or TPR) and precision (i.e., PPV) of VarDict, Mutect2, LoFreq and VarScan2, on samples containing 100 spiked SNVs each. Performances of the variant callers are evaluated using the high-sensitivity settings and are reported for both the DREAM challenge set 1 dataset (a), and 10 artificial samples generated with characteristics similar to those of the DREAM dataset (b).

References

Cibulskis, K., Lawrence, M.S., Carter, S.L., Sivachenko, A., Jaffe, D., Sougnez, C., Gabriel, S., Meyerson, M., Lander, E.S., & Getz, G. (2013). Sensitive detection of somatic point mutations in impure and heterogeneous cancer samples. *Nature Biotechnology*, 31(3), 213–219.

Ewing, A.D., Houlahan, K.E., Hu, Y., Ellrott, K., Caloian, C., Yamaguchi, T.N., Bare, J.C., P'ng, C., Waggott, D., Sabelnykova, V.Y., ICGC-TCGA DREAM Somatic Mutation Calling Challenge participants, Kellen, M.R., Norman, T.C., Haussler, D., Friend, S.H., Stolovitzky, G., Margolin, A.A., Stuart, J.M., & Boutros, P.C. (2015). Combining tumor genome simulation with crowdsourcing to benchmark somatic single-nucleotide-variant detection. *Nature Methods*, 12(7), 623–630.

Garrison, E., & Marth, G. (2012) Haplotype-based variant detection from short-read sequencing. *arXiv preprint arXiv:1207.3907 [q-bio.GN]*.

Kim, S., Scheffler, K., Halpern, A. L., Bekritsky, M. A., Noh, E., Källberg, M., Chen, X., Kim, Y., Beyter, D., Krusche, P., & Saunders, C. T. (2018). Strelka2: fast and accurate calling of germline and somatic variants. *Nature Methods*, 15, 591–594.

Koboldt, D.C., Zhang, Q., Larson, D.E., Shen, D., McLellan, M.D., Lin, L., Miller, C.A., Mardis, E.R., Ding, L., & Wilson, R.K. (2012). VarScan 2: somatic mutation and copy number alteration discovery in cancer by exome sequencing. *Genome Research*, 22(3), 568–576.

Lai, Z., Markovets, A., Ahdesmaki, M., Chapman, B., Hofmann, O., McEwen, R., Johnson, J., Dougherty, B., Barrett, J.C., & Dry, J.R. (2016). VarDict: a novel and versatile variant caller for next-generation sequencing in cancer research. *Nucleic Acids Research*, 44(11), e108.

Li, H., & Durbin, R. (2009). Fast and accurate short read alignment with Burrows-Wheeler transform. *Bioinformatics (Oxford, England)*, 25(14), 1754–1760.

Li, H., Handsaker, B., Wysoker, A., Fennell, T., Ruan, J., Homer, N., Marth, G., Abecasis, G., Durbin, R., & 1000 Genome Project Data Processing Subgroup (2009). The Sequence Alignment/Map format and SAMtools. *Bioinformatics (Oxford, England)*, 25(16), 2078–2079.

Pedersen, B.S., & Quinlan, A.R. (2017). cyvcf2: fast, flexible variant analysis with Python. *Bioinformatics (Oxford, England)*, 33(12), 1867–1869.

Stephens, Z.D., Hudson, M.E., Mainzer, L.S., Taschuk, M., Weber, M.R., & Iyer, R.K. (2016). Simulating Next-Generation Sequencing datasets from empirical mutation and sequencing models. PLOS One, 11(11), e0167047.

Tarasov A., Vilella A.J., Cuppen E., Nijman I.J., & Prins P (2015). Sambamba: fast processing of NGS alignment formats. *Bioinformatics*, 31(12), 2032-2034.

Wilm, A., Aw, P.P., Bertrand, D., *et al.* (2012) LoFreq: a sequence-quality aware, ultra-sensitive variant caller for uncovering cell-population heterogeneity from high-throughput sequencing datasets. *Nucleic Acids Research*. 40(22), 11189-11201.
